# Functional Antagonism of Junctional Adhesion Molecule-A (JAM-A), Overexpressed in Breast Ductal Carcinoma In Situ (DCIS), Reduces HER2-Positive Tumor Progression

**DOI:** 10.3390/cancers14051303

**Published:** 2022-03-03

**Authors:** Yvonne E. Smith, Guannan Wang, Ciara L. Flynn, Stephen F. Madden, Owen MacEneaney, Rodrigo G. B. Cruz, Cathy E. Richards, Hanne Jahns, Marian Brennan, Mattia Cremona, Bryan T. Hennessy, Katherine Sheehan, Alexander Casucci, Faizah A. Sani, Lance Hudson, Joanna Fay, Sri H. Vellanki, Siobhan O’Flaherty, Marc Devocelle, Arnold D. K. Hill, Kieran Brennan, Saraswati Sukumar, Ann M. Hopkins

**Affiliations:** 1 Department of Surgery, RCSI University of Medicine and Health Sciences, Beaumont Hospital, Dublin 9, Ireland; yvonnesmith_6@hotmail.com (Y.E.S.); ciaralouiseflynn@hotmail.com (C.L.F.); rodrigogbc@hotmail.com (R.G.B.C.); catherinerichards@rcsi.ie (C.E.R.); lhudson@rcsi.ie (L.H.); vellanki.sriharikrishna@gmail.com (S.H.V.); adkhill@rcsi.ie (A.D.K.H.); ciaran.brennan2@gmail.com (K.B.); 2 Department of Oncology, Johns Hopkins University School of Medicine, Baltimore, MD 21231, USA; gw288@georgetown.edu (G.W.); saras@jhmi.edu (S.S.); 3 Data Science Centre, RCSI University of Medicine and Health Sciences, Dublin 2, Ireland; stephenmadden@rcsi.ie; 4 Department of Pathology, RCSI University of Medicine and Health Sciences, Beaumont Hospital, Dublin 9, Ireland; owenmaceneaney@rcsi.ie (O.M.); ksheehan@rcsi.ie (K.S.); joannafay@rcsi.ie (J.F.); 5 School of Veterinary Medicine, University College Dublin, Dublin 4, Ireland; hanne.jahns@ucd.ie; 6 School of Pharmacy and Biomolecular Sciences, RCSI University of Medicine and Health Sciences, Dublin 2, Ireland; mbrennan3@rcsi.ie; 7 Department of Medical Oncology, RCSI University of Medicine and Health Sciences, Beaumont Hospital, Dublin 9, Ireland; mattiacremona@rcsi.ie (M.C.); bryanhennessy74@gmail.com (B.T.H.); 8 School of Medicine, RCSI University of Medicine and Health Sciences, Dublin 2, Ireland; alexandercasucci@gmail.com (A.C.); faizahabubakarsani@rcsi.ie (F.A.S.); 9 Department of Chemistry, RCSI University of Medicine and Health Sciences, Dublin 2, Ireland; siobhanoflaherty@rcsi.ie (S.O.); mdevocelle@rcsi.ie (M.D.)

**Keywords:** DCIS, junctional adhesion molecule-A (JAM-A), breast cancer, antagonism, intra-ductal, tight junction, in vivo

## Abstract

**Simple Summary:**

Specific drug targets for breast ductal carcinoma in situ (DCIS) remain elusive, despite increasing disease prevalence and burden to healthcare services. Estrogen receptor (ER)-negative HER2-positive DCIS, associated with the poorest patient prognosis, is in particular need of novel therapeutic avenues. This report provides the first evidence that a cell surface protein called JAM-A is upregulated on human DCIS patient tissues and can be readily targeted by a novel JAM-A-binding peptide inhibitor in separate in vivo models of DCIS. The anti-tumor efficacy and lack of systemic toxicity of this lead inhibitor, coupled with early indications of potential signaling pathways implicated, support the value of future studies investigating JAM-A as a novel drug target in DCIS patients.

**Abstract:**

Breast ductal carcinoma in situ (DCIS) is clinically challenging, featuring high diagnosis rates and few targeted therapies. Expression/signaling from junctional adhesion molecule-A (JAM-A) has been linked to poor prognosis in invasive breast cancers, but its role in DCIS is unknown. Since progression from DCIS to invasive cancer has been linked with overexpression of the human epidermal growth factor receptor-2 (HER2), and JAM-A regulates HER2 expression, we evaluated JAM-A as a therapeutic target in DCIS. JAM-A expression was immunohistochemically assessed in patient DCIS tissues. A novel JAM-A antagonist (JBS2) was designed and tested alone/in combination with the HER2 kinase inhibitor lapatinib, using SUM-225 cells in vitro and in vivo as validated DCIS models. Murine tumors were proteomically analyzed. JAM-A expression was moderate/high in 96% of DCIS patient tissues, versus 23% of normal adjacent tissues. JBS2 bound to recombinant JAM-A, inhibiting cell viability in SUM-225 cells and a primary DCIS culture in vitro and in a chick embryo xenograft model. JBS2 reduced tumor progression in in vivo models of SUM-225 cells engrafted into mammary fat pads or directly injected into the mammary ducts of NOD-SCID mice. Preliminary proteomic analysis revealed alterations in angiogenic and apoptotic pathways. High JAM-A expression in aggressive DCIS lesions and their sensitivity to treatment by a novel JAM-A antagonist support the viability of testing JAM-A as a novel therapeutic target in DCIS.

## 1. Introduction

Ductal carcinoma in situ (DCIS) is a pre-invasive form of breast cancer in which tumors are histologically confined to milk ducts without locally invading past the myoepithelial layer encircling the ductal epithelium, making them surgically resectable with high success rates [1]. Approximately 20% of breast cancer patients present with DCIS, frequently diagnosed via screening mammography. The widespread use of mammography has thus led to a rise in the incidence of DCIS [2]. Genomic and phenotypic similarities have suggested DCIS as a precursor of invasive ductal carcinoma [3]; however, multi-decade follow-up studies of DCIS patients whose only interventions were surgical biopsies have estimated that in fact <40% of patients develop invasive carcinoma traceable to the site of the initial DCIS lesion [4]. The presence of indolent disease for several decades in the remainder of patients, or rather the lack of progression of their disease into invasive ductal carcinoma, highlights an important clinical conundrum of how to distinguish between DCIS patients whose disease is likely to progress or not. Although much progress has been made in identifying genomic [5] and proteomic [6] signatures to help stratify risk, a deeper understanding of the biology of DCIS is still warranted to guide surgical best practice and the development of targeted therapies.

This study centers upon Junctional Adhesion Molecule-A (JAM-A), a transmembrane immunoglobulin superfamily protein with numerous physiological functions (reviewed by [7]), whose overexpression in invasive breast cancer patient tissues has been associated with aggressive or recurrent disease and increased risk of metastasis [8,9,10,11,12]. Evidence of JAM-A overexpression in multiple cancers is also accumulating [13]. Of specific interest for the current study is the fact that JAM-A expression is high in the human epidermal growth factor receptor-2 (HER2) genomic subtype of invasive breast cancer, and that JAM-A levels have been shown to influence those of HER2 at both transcriptional and translational levels in breast cancer cells in vitro [8,10]. A potential connection with JAM-A is of importance since high HER2 expression has been suggested to flag DCIS tumors that progress to invasive disease [14]; if JAM-A is indeed an upstream regulator of HER2, this might suggest its druggability in HER2-positive breast cancer. Studies have suggested therapeutic value in inhibiting JAM-A signaling in in vivo models of invasive breast cancer [9], but the precise mechanisms remain elusive. JAM-A may represent an attractive cancer target, however, given its cell surface localization and capacity to regulate multiple signaling events driving tumor progression (including proliferation, apoptosis, migration, angiogenesis, and cancer stem cell renewal; reviewed by [7]). However, the potential contribution of JAM-A overexpression to early-stage or pre-invasive breast cancer, like DCIS, is currently unknown. In this study we provide the first evidence that JAM-A is overexpressed in a high proportion of human DCIS breast tumors. Using a murine mammary fat pad xenograft model of human HER2-positive DCIS, a direct murine intra-ductal administration model [15], and a chick embryo xenograft model, we demonstrate that JAM-A functional antagonism using a novel JAM-A-binding peptide hinders tumor progression. Taken together with proteomic evidence of alterations in angiogenic and apoptotic pathways, our results suggest value in further in-depth testing of JAM-A as a novel therapeutic target in HER2-positive DCIS patients.

## 2. Materials and Methods

### 2.1. JAM-A Immunohistochemistry

A breast tissue microarray (TMA; US Biomax-BR8011a) containing 50 DCIS cases and 26 normal adjacent tissues (NAT) was immunohistochemically stained for JAM-A on a Bond-III immunostainer (Leica Biosystems, Newcastle, UK) as follows: antigen retrieval 20 min with Bond Epitope Retrieval Solution 1 (Leica Biosystems, Newcastle, UK), 1:1000 dilution of monoclonal antibody M01 clone 2E3-1C8 (Abnova, Taipei City, Taiwan). Detection and visualization of stained cells was achieved using the Bond Polymer Refine Detection Kit (Leica) with Bond DAB Enhancer (Leica). Tissues were counterstained with hematoxylin and cover-slipped. Cores were semi-quantitatively scored as described in [8]: 0 = no staining, 1 = weak membranous JAM-A positivity, 2 = membranous positivity in <10% of cells (termed moderately positive), 3 = membranous positivity in ≥10% of cells (termed strongly positive). Detailed patient information was not available from the company for this TMA.

### 2.2. Design, Molecular Modelling, and Synthesis of JAM-A-Interacting Peptide JBS2

A 5-mer peptide JBS2 (GETRG) was designed to bind to the JAM-A *cis*-dimerization site, based on crystallography data for murine JAM-A–JAM-A interactions [16] and JAM-A–reovirus interactions [17]. This site is spatially distinct from the JAM-A *trans*-dimerization site [18], which is thought to function in physiological adhesion. A 2-dimensional interaction map was generated using Molecular Operating Environment Software (MOE v.2013.08; Chemical Computing Group, Montreal, QC, Canada) to define interactions between JBS2 and the amino acids of human JAM-A. The protein was prepared using proteinate-3D and ligand interactions predicted using the ligand interaction function. Docking was performed using Triangle matcher according to default parameters. JBS2 was commercially synthesized (GenScript, Piscataway, NJ, USA; Peptide2.0, Chantilly, VA, USA) to >95% purity against a chloride counter-ion with guaranteed removal of trifluoroacetic acid. For the chick embryo xenograft model, peptide JBS2 was prepared locally by standard solid-phase peptide synthesis according to the Fmoc-tBu strategy. The synthesis was carried out from an Fmoc-Gly-Wang resin on an automated peptide synthesizer (CEM Liberty Blue™; CEM Microwave Technology Ireland Ltd., Damastown, Ireland) at 0.25 mmol scale. Trifluoroacetate to chloride exchange was performed by dissolving the peptide in water and treating it with an ion exchange resin (Dowex^®^ 1X8 chloride, Merck, Ireland) at RT, with monitoring by ^19^F-NMR in D_2_O. Chromatographic analysis and purification were performed on a Shimadzu Prominence HPLC system using Gemini columns (Phenomenex, 110 Å, 5 µ, C18, 4.6 mmd/250 mmL or 100 mmd/250 mmL for analytic or semi-preparative columns, respectively). Electrospray ionization mass spectroscopy (ESI-MS) analysis was performed with an Advion Expression CMS.

### 2.3. JBS2 Binding Assay

To begin, 96-well plates (Nunc Maxi-Sorp ELISA, BioLegend, San Diego, CA, USA) coated with 1 ng recombinant human JAM-A (ab151859, Abcam, Cambridge, UK; 6 h at 4 °C) were blocked overnight at 4 °C with 5% bovine serum albumin, incubated with increasing concentrations of *N*-terminally-biotinylated JBS2 (JPT Peptide Technologies GmBH, Berlin, Germany) or PBS (100 µL) control for 3 h at 37 °C, and washed once with phosphate-buffered saline/0.01% *v*/*v* Tween-20 (Sigma-Aldrich, Dharmstadt, Germany). Following incubation with horseradish peroxidase-coupled streptavidin (BioLegend, San Diego, CA, USA; 30 min, room temperature), three washes, and exposure to a buffered hydrogen peroxide substrate containing 10 mg O-phenylenediamine (1 h/37 °C), the reaction was stopped with 3 M HCl. Optical density at 490 nm was measured on a VICTOR™ X3 Multilabel Plate Reader (Perkin Elmer, Waltham, MA, USA) and was proportional to peptide binding levels.

### 2.4. Cell Culture

SUM-225-*luc* breast cancer cells [19] (gift of S. Ethier, Medical University of South Carolina) were cultured in DMEM/Ham’s F12 medium supplemented with 5% *v*/*v* fetal bovine serum, 2 mM L-glutamine, 50 U/mL penicillin, 50 µg/mL streptomycin, 10 µg/mL insulin, and 50 µg/mL hydrocortisone in a humidified incubator with 5% CO_2_. Primary cells were isolated from a 58-year-old female breast cancer patient with high-grade DCIS (with informed consent and ethical approval from the Beaumont Hospital Medical Ethics (Research) Committee, approval 07/74) as previously described [20]. For viability assays, 2 × 10^3^ cells/well were seeded in 96-well plates and treated a day later with JBS2 (200–400 µg/mL) or vehicle (PBS without calcium or magnesium 40% *v*/*v*) for 144 h in antibiotic-free medium (with medium change and fresh treatment at 72 h). Cells were incubated for the final 5 h with 0.5 mg/mL 3-(4,5-dimethylthiazol-2-yl)-2,5-diphenyl tetrazolium bromide (MTT, protected from light), and the formazan product was solubilized with 200μL dimethylsulfoxide (5 min/37 °C). Absorbance was measured at 560 nm using a VICTOR™-X3-Multilabel Plate Reader. For reverse-phase protein arrays, SUM-225 cells (1 × 10^5^/well) were plated in duplicate in 6-well plates and treated for two consecutive 72 h periods with JBS2 (400 µg/mL), the estimated IC_50_ of lapatinib in these cells (0.1 µM/L; Supplemental Figure S1A), JBS2 plus lapatinib, or vehicle controls (PBS 20% *v*/*v* for JBS2 control; DMSO 0.9% *v*/*v* for lapatinib control; PBS 20% *v*/*v* and DMSO 0.9% *v*/*v* for JBS2+lapatinib control). Lapatinib was chosen as the positive control anti-HER2 therapy rather than the inhibitory antibody trastuzumab/Herceptin, since the latter was predictably ineffective under purely in vitro conditions.

### 2.5. Mammary Fat Pad (mfp) Murine Model of Breast Cancer

With ethical approval from the Irish Health Products Regulatory Agency under Directive 2010/63/EU (AE19127_P015), sixty 9-to-13-week-old female NOD-SCID mice (NOD.CB17-*Prkdc^scid^*/NCrCrl, Charles River, Margate, UK) were orthotopically implanted with 1 × 10^7^ SUM-225-Luc+ cells in a 1:1 volume of Matrigel into the fourth left inguinal mfp. This was based on power calculations (using http://www.biomath.info, accessed 16 July 2015) following a pilot study with a different JAM-A antagonistic peptide in another mouse model, which estimated that 15 animals per treatment group would be required to see statistically significant reductions of 50% in tumor size. Over an 8-to-15-week period, 33 mice achieved tumors with the desired volume of ≥100 mm^3^ (measured by calipers, where volume = tumor width^2^ × length/2), and 27 did not. The 33 animals with tumors ≥100 mm^3^ were randomized (using a random number generator online tool) into one of four treatment groups. These were treated for 28 days as follows: PBS (*n* = 8; daily intra-peritoneal/i.p. injection at equivalent volume to JBS2, typically 120–150 µL), JBS2 (*n* = 9; 10 mg/kg daily i.p), lapatinib tosylate (Carbosynth Ltd., Berkshire, UK; *n* = 8; 100 mg/kg oral gavage on 5 consecutive days per week), JBS2 plus lapatinib (*n =* 8; 10 mg/kg and 100 mg/kg respectively). Tumor volumes were measured twice-weekly using calipers, and tumor bioluminescence was measured once-weekly using the In Vivo Imaging System (IVIS, Perkin Elmer, Waltham, MA, USA) following subcutaneous injection with 150 mg/kg D-luciferin. Five animals were sacrificed early for treatment-unrelated pathologies (4 lymphomas and 1 rectal prolapse) and excluded from the analysis (2 PBS, 1 JBS2, 2 lapatinib). Tumors were excised on day 28, whereupon one half was snap-frozen in liquid nitrogen and stored at −80 °C, and the other half was fixed in 10% (*v*/*v*) neutral-buffered formalin. Sections of 4 µm were immunohistochemically stained for human JAM-A or cytokeratin-5/6 (Dako M737 Clone D5/16-B4, 1:50, Bond Epitope Retrieval Solution 2 (Leica)).

### 2.6. Murine Intraductal Model of DCIS

Following protocols approved by the Animal Care and Use Committee of Johns Hopkins Medical Institutions, 0.5 × 10^5^–1 × 10^5^ SUM-225-*luc* cells were intraductally injected into the fourth pair of mammary glands of multiparous female NOD-SCID-gamma (NOD.Cg-*Prkdc^scid^ Il2rg^tm1Wjl^*/SzJ) ex-breeder mice bred at Johns Hopkins, essentially as described in [15]. Power calculations were not performed in advance, as this was a pilot observational study. Animals were injected intraductally with 10 mg/mL JBS2 in 20 µL PBS, or PBS alone, on days 7, 14, and 21 after cell injection. IVIS imaging was performed on days 7 (baseline), 14, 21, and 28 post implantation. Tumors were excised on day 28, and then formalin-fixed and paraffin-embedded (FFPE).

### 2.7. Reverse-Phase Protein Array (RPPA) Analysis

Total protein extracts were prepared from snap-frozen SUM-225 mfp tumors or SUM-225 cell cultures (treated for 144 h as described) in lysis buffer (1% Triton X-100, 50 mM HEPES pH 7.4, 150 mM NaCl, 1.5 mM MgCl_2_, 1 mM EGTA, 100 mM NaF, 10 mM Na pyrophosphate, 1 mM Na_3_VO_4_, 10% glycerol, containing freshly added protease and phosphatase inhibitors (Sigma-Aldrich, Dharmstadt, Germany)) and adjusted to a concentration of 1.5 mg/mL. The lysate was resuspended in 4× SDS sample buffer without bromophenol blue and processed for RPPA (blinded to the operator) against a panel of 55 primary antibodies (Supplemental Table S1), as described in [21]. Spots were normalized by protein loading using the entire panel of antibodies. Briefly, the normalization was as follows: Median values were determined for each antibody across the sample set, and each raw linear value was divided by the median within each antibody to get the median-centered ratio. The median from the median-centered ratio was then calculated for each sample across the entire panel of antibodies. This median functions as a correction factor (CF) for protein-loading adjustment. Samples are considered to be outliers if the CF is above 2.5 or below 0.25. Finally, the raw data (in linear values) was divided by the CF to obtain the normalized value.

### 2.8. Multiplex Protein Array Profiling in Tissues

Total protein extracts were prepared from snap-frozen SUM-225 tumors grown in the mfp as described for RPPA, or from 10 × 5 µm FFPE tissue sections from SUM-225 intraductal tumors with modifications of established protocols [22]. Deparaffinized sections were treated in 2% SDS and heated (95 °C/20 min and 70 °C/2 h) with shaking. Proteins were quantified (blinded to the operator) using Olink multiplex proximity extension assay (PEA; Olink Proteomics; www.olink.com, last accessed 20 January 2022; Oncology-II panel, 92 biomarkers) as described elsewhere [23]. Briefly, PEA technology utilized two matched antibodies labelled with unique DNA oligonucleotides that simultaneously bound their target protein in solution, bringing the antibodies into proximity and allowing their oligonucleotides to hybridize and serve as a template for DNA polymerase-dependent extension. The amount of antigen-unique double-stranded DNA “barcode” was quantitatively proportional to the initial concentration of target protein and was detected by PCR amplification and amplicon quantification by microfluidic qPCR (Fluidigm BioMark HD system; Fluidigm Corporation). The resulting Ct-data was then quality controlled and normalized using a set of internal and external controls. The final assay read-out was presented in Normalized Protein eXpression (NPX) values, an arbitrary unit on a log2-scale where high values correspond to high protein expression. The internal controls were designed to mimic and monitor the different steps of the PEA. They consisted of two incubation/immuno controls, an extension control, and a detection control. Internal controls were introduced to all samples, as well as to the external controls, and were used for quality control and data normalization. The external controls consisted of a negative control used to calculate the limit of detection (LOD), and a triplicate of interplate controls used for data normalization. Quality control of the data was performed in two steps: Firstly, runs were quality controlled by calculating the standard deviation (sd) for the detection control and the incubation/immuno controls (where sd > 0.2 failed quality control). Secondly, each sample was quality controlled by comparing the results for the detection control and one of the incubation controls against the run median. Samples that fell >0.3 NPX from the run median relative to these two internal controls failed quality control. All assay validation data (detection limits, intra- and inter-assay precision data, etc.) are available on the manufacturer’s website (www.olink.com, last accessed 20 January 2022). Antibodies used are listed in Supplemental Table S2. 

### 2.9. Chorioallantoic Membrane (CAM) Chick Embryo Xenograft Model

Fertilized chicken eggs (Shannonvale Hatchery, Limerick, Ireland) were incubated at 37 °C, and on gestation day 3, a window was opened, and fluid withdrawn, to lower the CAM. On day 9, 2 × 10^6^ SUM-225 cells resuspended 1:1 in 100% (*v*/*v*) Matrigel were added to the CAM within a silicon ring. Xenografts were treated with PBS (15 µL), JBS2 (200 µM), lapatinib (0.1 µM), or JBS2+lapatinib on days 9 and 13. On day 14, embryos were sacrificed, and xenografts were isolated and fixed in formaldehyde (4%/overnight). They were washed with 70% ethanol, paraffin-embedded, and stained with hematoxylin/eosin or immunohistochemically for the proliferation marker Ki67 and CK5/6, as described in [10]. 

### 2.10. Statistical Analysis

A Fisher’s exact test was used to compare the proportion of DCIS tumors and normal adjacent breast tissues with moderate/high JAM-A expression. One- or two-way ANOVAs (with/without repeated measures, as appropriate) were used to test JBS2 binding, its effects on cell viability, the effects of different drug combinations on tumor growth over time in the mfp model, tumor bioluminescence, or CK5/6 positivity in the mfp model. A mixed-effects model was used to test the effect of JBS2 on tumor growth in the mfp model. Post-hoc testing and *p*-value adjustments were performed using either Tukey’s honest significant difference (Tukey’s HSD) or a *t*-test followed by *p*-value adjustment using the Benjamini–Hochberg method [24]. A chi-squared test was used to evaluate the effect of JBS2 on gross tumor visibility and embryonic death in the chick embryo xenograft model. One-tailed unpaired Student’s *t*-tests were used to determine if JBS2 lowered JAM-A expression in mfp tumor sections, lowered tumor bioluminescence at individual timepoints in the i.duc model, or increased/decreased the expression of individual protein targets in the O-Link proteomics array conducted on tumor tissues from the i.duc model.

## 3. Results

### 3.1. JAM-A Expression Is Elevated in DCIS

**Figure 1:**
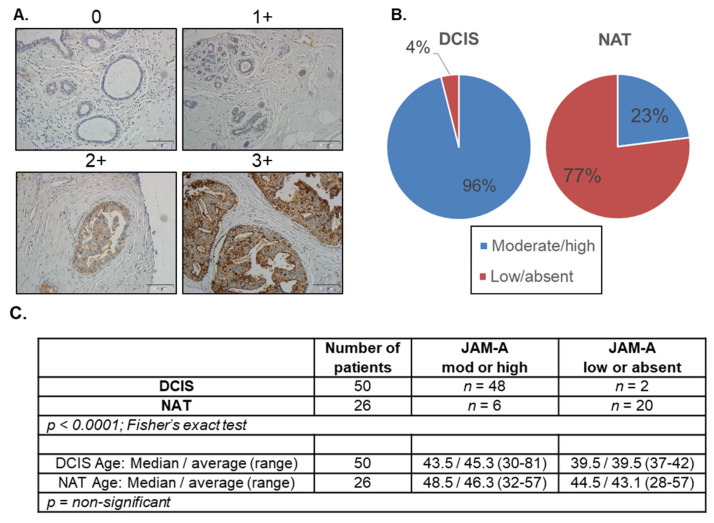
JAM-A expression in DCIS tumors exceeds that in normal breast tissue. (**A**) Membranous JAM-A expression was semi-quantitatively scored following immunohistochemical staining of a tissue microarray of DCIS patient cores or breast normal adjacent tissue (NAT). Representative images of 0 and 1+ scores were generated from NAT cores, while representative images of 2+ and 3+ scores were generated from DCIS cores (all 20× magnification). (**B**) JAM-A expression was noted as moderate/high (2+/3+) in 96% of DCIS cores compared to 23% of NAT cores (*p* < 0.0001). JAM-A staining of 0/1+ was categorized as absent/low. No trends in relation to patient age (**C**) were identified in this small sample set.

With evidence that high expression of the adhesion protein JAM-A marks aggressive disease in patients with invasive breast ductal carcinoma [8,9,10,11,12], this study set out to determine if JAM-A expression was also elevated at the earlier phase of DCIS. Immunohistochemical staining and semi-quantitative scoring (Figure 1A) of DCIS patient cases (*n* = 50) revealed that 96% of DCIS cores had moderate/high JAM-A expression versus only 23% of normal adjacent tissue cores (*n* = 26 patients; Figure 1B; *p* < 0.0001). However, there were no age-associated trends identified in this small cohort (Figure 1C), nor any available data on HER2 expression status. Of four lobular carcinoma in situ (LCIS) cores in the same TMA, 3/4 had moderate/high JAM-A staining, while the fourth was negative (see representative low/high power micrographs, Supplemental Figure S2). 

### 3.2. Designing a Peptide Antagonist to JAM-A

**Figure 2:**
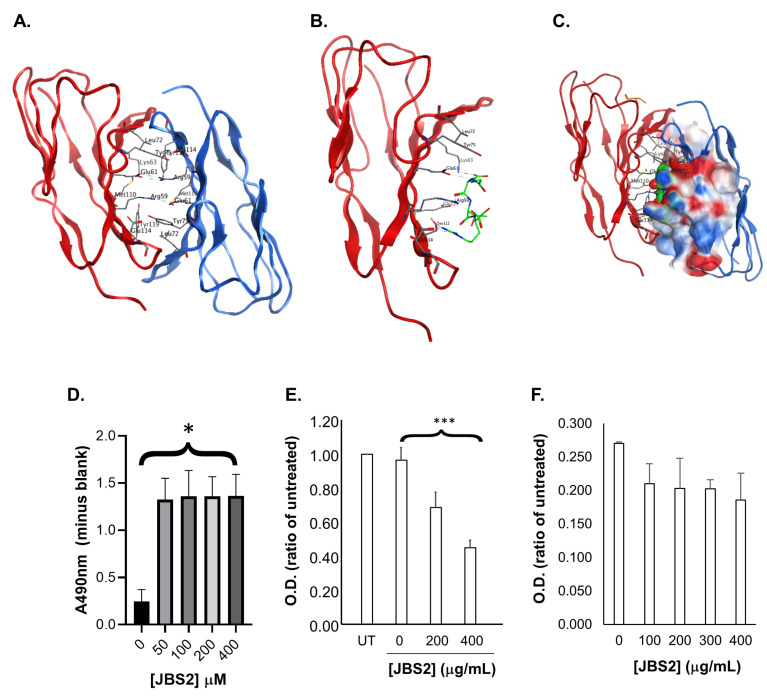
Peptide JBS2 binds to JAM-A and exerts anti-viability effects in vitro. (**A**) Crystal structure of the *cis*-dimer interface. Amino acid residues at the interface are displayed as sticks and colored according to element: oxygen is colored red, carbon grey, nitrogen blue, and serine yellow. (**B**) depicts the predicted binding pose for JBS2. The carbon atoms on the JBS2 peptide are colored in green. (**C**) presents an overlay of the JAM-A dimer and the predicted JBS2 binding pose. JBS2 is presented using space-fill with carbons colored in green. JBS2 was docked into the monomer with the red-colored ribbon. The surface of the interacting monomer is colored according to electrostatics (red, negative; blue, positive; and white, neutral). (**D**) Recombinant human JAM-A (1 ng) coated onto 96-well plates successfully captured biotinylated JBS2 peptide (50–400 mM; ~3–207 mg/mL) relative to wells without peptide (0 mM JBS2). Data represent mean ± SEM of *n* = 3 independent biological replicate experiments, and binding effects were concentration-independent (* *p* < 0.05). (**E**) SUM-225 cells grown on 96-well plates and treated for 2 × 72 h periods with the indicated concentrations of JBS2 or vehicle versus untreated cells (UT) were subjected to MTT viability assays. JBS2 exerted significant concentration-dependent reductions in cell viability. Data represent mean ± SEM of *n* = 3 independent biological replicate experiments; *** *p* < 0.001. (**F**) Primary breast cells isolated from a patient with high-grade DCIS were grown on 96-well plates, treated for 2 × 72 h periods with the indicated concentrations of JBS2 or vehicle, and subjected to MTT viability assays. Values shown are mean ± standard deviation of duplicate technical replicates in a single representative experiment. There was a trend towards reduced cell viability with JAM-A antagonism.

Since *cis*-dimerization of JAM-A reportedly drives its participation in functional behaviors associated with tumorigenesis [25], molecular modelling was used to design a peptide antagonist of JAM-A *cis*-dimerization (JBS2). The cis-dimer interaction of JAM-A is depicted in Figure 2A with residues at the interface displayed and the important ARG59 and GLU61 bonding interactions visible. The predicted binding pose for the five-mer peptide JBS2 docked into the JAM-A dimerization site (in the first extracellular Ig loop of JAM-A, most distal from the cell membrane) is presented in Figure 2B. JBS2 is predicted to form hydrogen bonding interactions with JAM-A residues GLU61 and SER112 and hydrogen bonding interactions with ARG59, LYS63, and GLU114. These amino acids have been identified as the residues involved in H-bond formation necessary to form the stable JAM-A dimer [16,17]. Figure 2C depicts an overlay of the interacting dimer with the docking result for JBS2. JBS2 is predicted to bind in the area that is occupied by the protein when JAM-A forms a *cis*-dimer and to be buried in the surface of the interacting monomer, indicating that while binding, it would sterically inhibit the dimer interaction. Thus, our results suggest that JBS2 can compete for the dimerization site, sterically blocking interaction, thereby preventing dimer formation. This was experimentally verified as being concentration-independent in a binding assay on recombinant purified JAM-A (Figure 2D). Functionally, JBS2 exerted concentration-dependent reductions in the viability of estrogen receptor (ER)-negative HER2-positive SUM-225 breast cancer cells, a DCIS-like cell model (Figure 2E), and it showed a trend to reduce viability in a primary culture from a DCIS patient (Figure 2F; *p* = ns). The bioactivity of JBS2 was not limited to HER2-positive cells, since JBS2 also significantly reduced cell viability in the JAM-A-expressing HER2-negative breast cell line MCF7 (Supplemental Figure S3A), in parallel with reductions in both AKT and ERK phosphorylation, without any effect on levels of either total AKT or total ERK (Supplemental Figure S3B). HER2-positive SUM-225 cells also responded in a concentration-dependent fashion to the positive control anti-HER2 drug lapatinib, with a calculated IC50 of 0.1 µmol/L (Supplemental Figure S1A), while the combination of JBS2 + lapatinib significantly attenuated cell viability over either drug alone (Supplemental Figure S1B). 

### 3.3. JAM-A Antagonism Reduces Tumor Growth In Vivo

**Figure 3:**
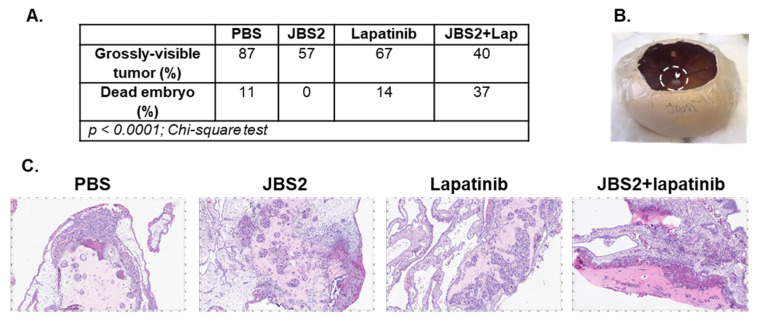
JAM-A antagonism reduces tumor size in a chick embryo xenograft model. (**A**) SUM-225 cells (2 × 10^6^) were implanted onto the chorioallantoic membrane overlying a developing chick embryo and treated then and 4 days later with 15 µL PBS, 200 µM JBS2, and 0.1 µM lapatinib or JBS + lapatinib. (**A**) Upon xenograft harvesting, the number of grossly visible tumors was observed to be less in treated relative to control conditions according to the following hierarchy: PBS > lapatinib > JBS2 > JBS2 + lapatinib (*n* = 5–8 eggs per condition). No embryonic death was observed for JBS2-treated eggs, while there were 1–3 embryonic deaths in the other conditions as follows: PBS < lapatinib < JBS2 + lapatinib (*** *p* < 0.0001). (**B**) Representative image of a gross xenograft tumor (arrowhead) before harvesting from the CAM. The boundary of the silicon ring is shown as a dashed line. (**C**) Representative images of formalin-fixed paraffin-embedded xenograft tumor sections (4 μm) stained with hematoxylin/eosin.

To begin interrogating the properties of JBS2+/−lapatinib in higher-order settings, an in vivo chick embryo xenograft model [26] was employed. JBS2 reduced the number of macroscopically visible tumors that grew on the chorioallantoic membrane (CAM) (Figure 3A), to a greater extent than lapatinib alone and a lesser extent than JBS2+lapatinib. However, JBS2 did not cause embryonic death, in contrast to lapatinib alone or particularly JBS2+lapatinib treatment (Figure 3A). A representative gross image of a xenograft tumor before excision from the CAM is shown in Figure 3B (white arrowhead; silicon ring annotated as dashed line). Haematoxylin and eosin staining of excised xenografts showed histologically similar tumors, where viable tumor remained (Figure 3C). 

**Figure 4:**
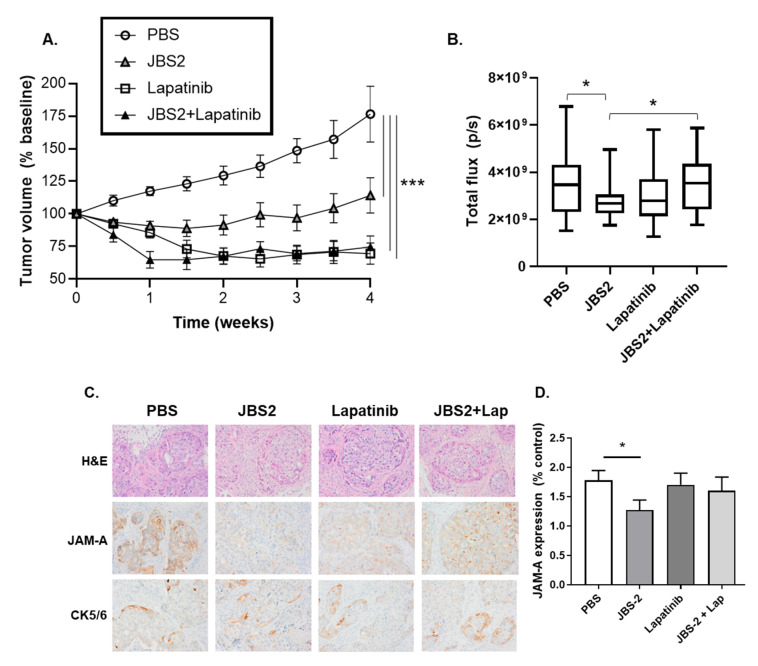

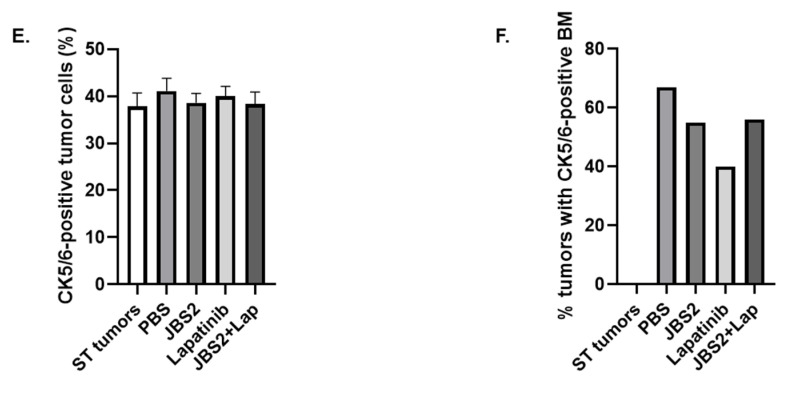
JAM-A antagonism reduces tumor progression in an mfp tumor xenograft model. (**A**) SUM-225-*luc* tumor xenografts grown to a volume of 100 mm^3^ in the mfps of NOD/SCID mice were treated for 4 weeks with PBS, JBS2, lapatinib, or JBS2 + lapatinib and volumetrically measured twice/week (*n* = 6, 9, 6, 8 mice, respectively). JBS2, lapatinib, and JBS2+lapatinib all significantly reduced tumor growth over time versus PBS-treated tumors (*** *p* < 0.001). Co-administration of JBS2 with the positive control drug lapatinib significantly shortened the time until lapatinib reached its maximal effect (* *p* < 0.05, comparing volumes in lapatinib versus JBS2+lapatinib tumors at 1 week post treatment). (**B**) Tumor bioluminescence was imaged once-weekly by in vivo imaging system (IVIS), and mean values over the entire treatment period (weeks 1–4 inclusive) expressed as flux (pixels/second; p/s) were plotted. Graph shows maximum and minimum values with a line at the mean bioluminescence for each treatment group. JBS2 significantly reduced mean tumor bioluminescence (* *p* < 0.05; PBS versus JBS2 and JBS2 versus JBS2 + Lapatinib). (**C**) FFPE tumor sections were stained with hematoxylin and eosin (top panel) or immunohistochemically assessed for the expression of JAM-A (middle panel) or cytokeratin (CK)-5/6 (lower panel). (**D**) JAM-A expression (measured semi-quantitatively) was reduced in tumor sections from mice treated with JBS2 relative to PBS (* *p* < 0.05). (**E**) The percentage positivity for cytokeratin 5/6 expression in xenograft tumor cells was not altered between treatment groups (*n* = 9–11 mice per group), where ST refers to sub-threshold tumors that never reached 100 mm^3^ and were thus not randomized to receive treatment. All treatments reduced the percentage of CK5/6-positive cells in xenograft basement membranes (BM); (**F**) a surrogate for tumor cell invasion.

An in vivo orthotopic mouse model of early breast cancer was next generated by injecting SUM-225-luc cells into the mfp of female NOD-SCID mice. Over the course of several weeks, these developed intra-ductal tumors resembling high-grade DCIS with comedo-necrosis and microinvasion, with high protein expression levels of both JAM-A and HER2 (Supplemental Figure S4). Tumors that reached a volume of ≥100 mm^3^ were randomized for 28-day treatment with PBS, JBS2, lapatinib, or JBS2 + lapatinib. As demonstrated in Figure 4A, control (PBS) tumors exhibited a gradual linear increase in tumor volume (measured twice-weekly by calipers). This was significantly attenuated by JBS2, lapatinib, or JBS2 + lapatinib (Figure 4A, *** p < 0.001). While the positive control HER2 kinase inhibitor lapatinib was more effective than JBS2 at reducing tumor volume, it is noteworthy that co-treatment with lapatinib+JBS2 significantly shortened the time for lapatinib to reach its maximal effect, from 2 weeks to 1 week (Figure 4A, * *p* = 0.026 comparing JBS2+lapatinib versus lapatinib alone after 1-week treatment). 

Analysis of weekly bioluminescence data revealed no simple relationship paralleling the volumetric measurements from Figure 4A. This may reflect falsely high values from necrotic tumors. Therefore, the data were represented as the mean of all bioluminescence measurements within each treatment group over the entire treatment period (weeks 1–4). As per Figure 4B, bioluminescence measurements confirmed that JBS2 significantly reduced mean tumor size relative to PBS-treated tumors (Figure 4B, * *p* < 0.05). The mean bioluminescence value of lapatinib was similar to that of JBS2, but this was not statistically significant. Interestingly, mean tumor bioluminescence for JBS2/lapatinib was similar to PBS controls and significantly exceeded that of JBS2 alone (Figure 4B). When tumors were stained for JAM-A and cytokeratin-5/6 (Figure 4C), quantification revealed that JBS2-treated tumors had significantly less JAM-A (Figure 4D) but not cytokeratin-5/6 expression (Figure 4E). However, the percentage of tumors with cytokeratin-5/6 positivity in the basement membrane (BM; a surrogate for tumor invasion) was reduced by treatment with either JBS2, lapatinib, or JBS2 + lapatinib (Figure 4F). In comparison, of nine sub-threshold (ST) tumors that did not reach a volume of 100 mm^3^ (and thus were not randomized into treatment groups), 100% were negative for cytokeratin-5/6 expression in the BM. 

### 3.4. Proteomic Changes in JAM-A-Antagonized Tumors

A selection of tumors from mice that had completed 28 days of treatment were subjected to reverse-phase protein array (RPPA) proteomics to investigate targets altered alongside drug-induced inhibition of tumor progression. Although lapatinib treatment showed enough significant changes in HER2 pathway target proteins to validate the approach (Supplemental Figure S5), drug treatments induced a mixed response, with both enhancements and reductions in expression of pro-tumorigenic proteins (cRAF, phospho-p38MAPK T180, S6 ribosomal protein, phospho-PDK1 S241 (Supplemental Figure S6)) in parallel with decreased levels of the pro-apoptotic protein HIAP2 (Supplemental Figure S6). These targets did not change in an RPPA performed on SUM-225 cells cultured in vitro for 6 days with the same treatments (Supplemental Figure S7; *n* = 3 independent experiments). In that setting, the only significant expressional changes were in Y527-phosphorylated-Src (increased in JBS2 + lapatinib-treated cells versus all other conditions) and Bcl-XL (decreased in JBS2 + lapatinib-treated cells compared with lapatinib or PBS) (Supplemental Figure S7).

**Figure 5:**
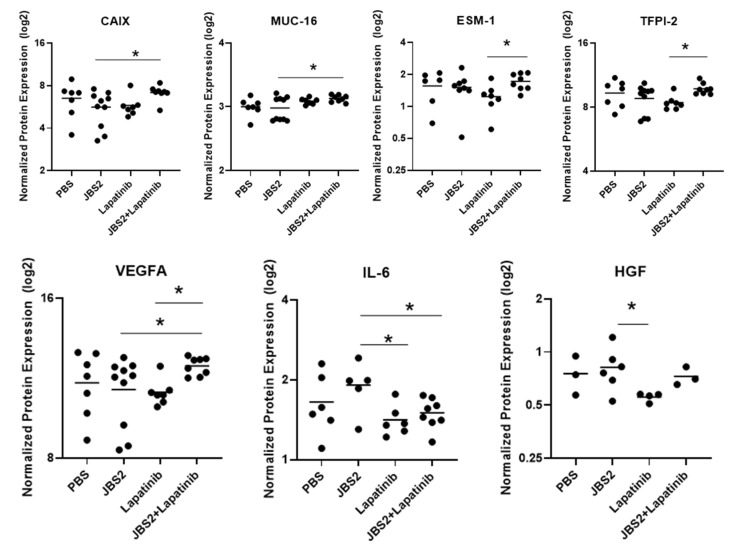
JAM-A and HER2 antagonism evoke multiple proteomic changes in mfp tumor xenografts. Fresh-frozen tissues harvested from the SUM-225 mouse mfp model of breast cancer (treatment groups: PBS, JBS2, lapatinib, JBS2+lapatinib) were subjected to proximity ligation assay proteomic analysis using an oncology-centered 92-protein array panel. Seven individual protein targets changed significantly between conditions in the 92-protein array (CAIX, MUC-16, ESM-1, TFPI-2, VEGFA, IL-6, HGF; * *p* < 0.05).

Proximity ligation assay technology was next utilized as an alternative screening tool to rank the most promising proteomic results from the mfp model for future study (Figure 5). Statistically speaking, a cut-off *p*-value < 0.05 was chosen for ranking, followed by individual one-way ANOVAs and the Benjamini–Hochberg method, recognizing that these would fail using a multiple ranking approach. Co-treatment with JBS2+lapatinib significantly increased the protein expression of carbonic anhydrase IX (CAIX) and Muc-16 (versus JBS2 alone) and increased that of ESM-1 and TFPI-2 (versus lapatinib alone) (Figure 5). Furthermore, lapatinib+/−JBS2 reduced the expression of IL-6 (versus JBS2 alone), and lapatinib + JBS2 increased that of VEGFA (versus either JBS2 or lapatinib). Finally, lapatinib reduced the expression level of hepatocyte growth factor (versus JBS2).

**Figure 6:**
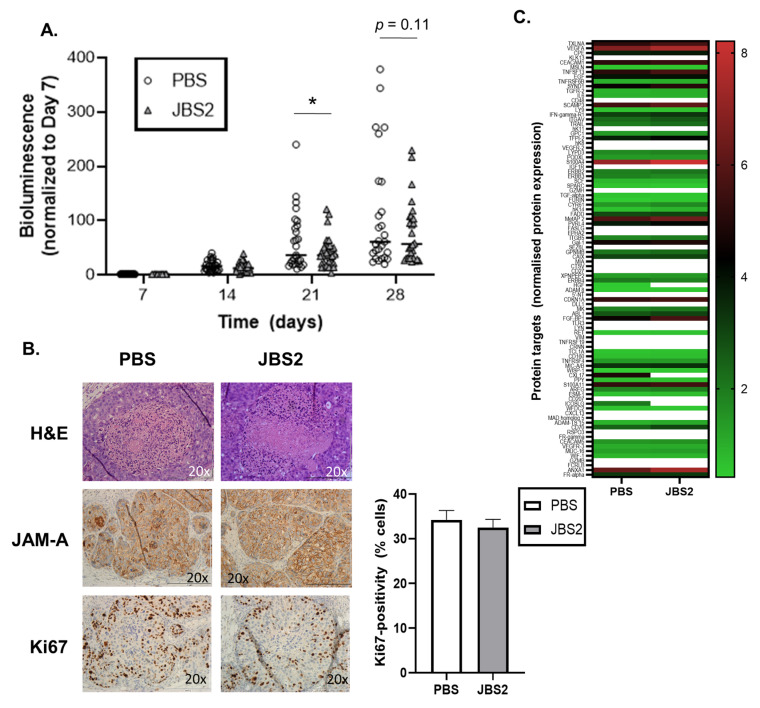

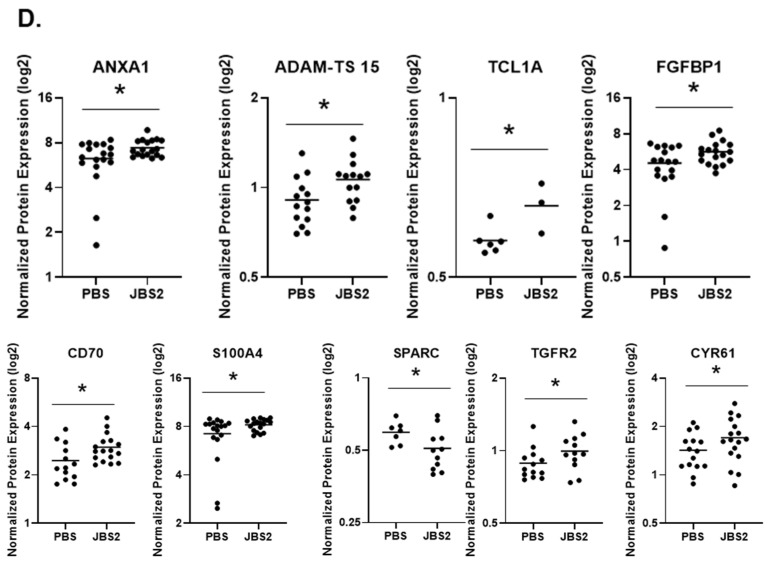
JAM-A antagonism reduces tumor progression and alters proteomic signaling in an intraductal murine cancer model. (**A**) DCIS tumors formed by direct intraductal injection of SUM-225-*luc* cells into NOD-SCID mice were treated once weekly for 3 weeks with JBS2 (0.2 mg) or PBS (20 µL), and their bioluminescence was measured weekly by IVIS. JBS2 reduced tumor bioluminescence at weeks 3 and 4 (* *p* < 0.05 at week 3). (**B**) FFPE sections stained with H&E showed intraductal tumors resembling high-grade DCIS with comedo-necrosis and microinvasion, which were morphologically similar in both treatment groups (upper panel). Immunohistochemistry for JAM-A showed similar expression in the JBS2-treated group (middle panel). No differences in Ki67 expression were identified between groups (lower panel and graph). (**C**) Protein extracts from FFPE tissues were subjected to proximity ligation assay proteomic analysis using an oncology-centered 92-protein array panel. Protein expression heatmap for the 92-protein array. (**D**) Nine individual protein targets changed significantly between PBS and JBS2 in the 92-protein array (* *p* < 0.05; annexin A1, ADAM-Thrombospondin-15, TCL1A, FGFBP1, CD70, S100A4—two-tailed unpaired *t*-test; SPARC, TGFR2, CYR61—one-tailed unpaired *t*-test).

In the absence of definitive protein signaling signatures induced by JBS2 in mfp DCIS tumors, the drug was functionally tested in an independent in vivo model of DCIS. SUM-225-luc cells were injected intraductally into NSG mice, followed by direct intraductal injection of JBS2 or PBS on days 7, 14, and 21 in parallel with IVIS imaging on those days, as well as day 28. As shown (Figure 6A), tumor bioluminescence in JBS2-treated mice was less than that in control mice on days 21 and 28 (after, respectively, 2 and 3 once-weekly intraductal injections; * *p* < 0.05 on day 21, PBS versus JBS2). As observed, all tumors appeared to have high-grade DCIS with comedo-necrosis, moderate-strong JAM-A staining, and similar levels of Ki67-positivity (Figure 6B). Representative FFPE tumor extracts from both treatment groups on day 28 were next compared via proximity ligation proteomics. Although formalin fixation was not compatible with the detection of several proteins (Figure 6C, white lines), statistically significant differences between treatment groups were nonetheless noted for nine proteins (Figure 6D). Encouragingly, JBS2 treatment upregulated levels of the pro-apoptotic/anti-inflammatory protein annexin A1 (ANXA1) and the tumor suppressor ADAM-TS-15 and downregulated the EMT factor SPARC. However, by day 28 (at which time bioluminescence changes between control and treated groups had lost their statistical significance), JBS2 had also upregulated levels of the proto-oncogene TCL1A; the pro-angiogenic factors FGFBP1, CYR61, and S100A4; the co-stimulatory molecule CD70; and the EMT factor TGFR2.

## 4. Discussion

Despite initial controversy over expression levels and roles of the adhesion protein JAM-A in invasive breast cancer [27], the balance of evidence has favored correlations between JAM-A overexpression and poor patient prognosis [8,10,11,12,13]. This study reports for the first time that high expression of JAM-A is also a prominent feature of DCIS early-stage breast cancer. Although echoing reports of JAM-A protein upregulation by the in situ stage in adenocarcinomas of the cervix [28] and lung [29], it does not by itself provide evidence of a causal role for JAM-A in disease progression. However, in conjunction with evidence that JAM-A may regulate HER2 expression [8] and that HER2 expression is proportionally higher in DCIS than invasive ductal carcinomas [30] or flags DCIS tumors that progress to invasive disease [14], the aim of this study was to explore the value of pharmacologically inhibiting JAM-A in HER2-positive breast DCIS models.

A novel peptide (JBS2) designed against the *cis*-dimerization site [25] of JAM-A was confirmed to bind to recombinant human JAM-A and to lower the viability of ER-negative HER2-positive SUM-225 cells, HER2-negative MCF7 cells, and a patient DCIS primary culture in vitro. That the primary culture was less sensitive to JBS2 than the immortal cells likely reflects much slower proliferative rates in primary cells, giving less scope to observe functional effects upon drug treatment. It would have been desirable to increase the number of DCIS patient primary cultures used for analysis, but this was complicated by the fact that presentation with pure DCIS was uncommon in our hospital (patients usually had a combination of DCIS and invasive ductal carcinoma at the time of primary surgery). More importantly, in the current work, JBS2 reduced measures of tumor progression in three separate in vivo models utilizing SUM-225 cells, which have been demonstrated to form tumors in the mfp that are histologically indistinguishable from human DCIS [31,32]. Independence from exogenous estrogen is unsurprising given the ER-negativity of SUM-225 cells, but an asset in the sense that slow-growing murine tumors of this nature may better represent indolent DCIS tumors than models requiring supplementation with supraphysiological estrogen levels. Furthermore, since coincident ER-negativity/HER2-positivity in DCIS patients has been associated with larger tumors and increased recurrence rates [33], in addition to cancer stem cell enrichment [34], the subset of DCIS patients represented by SUM-225 cells has greater need of new drug targets than the majority of DCIS patients with low-risk ER-positive disease. 

There have been positive indicators that JAM-A is readily targetable in in vivo models by either inhibitory antibodies [9] or peptides [35]. In the present study, JBS2 was well tolerated, with no adverse impact on animal welfare in two mouse models and one chick embryo xenograft model. This likely reflects the fact that JBS2 was designed to target pathophysiological JAM-A *cis*-dimerization rather than physiological *trans*-dimerization, which contributes to assembling and maintaining biological barriers [18]. However, in the two models that tested co-administration of JBS2 with lapatinib, some unusual results were noted. Specifically, although JBS2/lapatinib co-treatment exerted the greatest reduction in physical tumor volume in a mouse mfp model, parallel bioluminescence data suggested the reverse. The unexpectedly high bioluminescence in JBS2/lapatinib co-treated animals may reflect high levels of tumor necrosis, consistent with our observation that chick embryo death was higher in eggs co-treated with JBS2/lapatinib relative to either agent alone. It is also noteworthy that coincident JAM-A/HER2 antagonism exerted a better additive effect in vitro than in vivo, likely reflecting the fact that lapatinib in vivo was already so potent that there was minimal room to observe any additional effects. Future studies may test whether JAM-A antagonism can potentiate the anti-tumor effects of lower concentrations of anti-HER2 drugs like lapatinib. It is also intriguing that JBS2 was more effective than lapatinib in the chick embryo model relative to the mouse mammary fat pad model, whereas lapatinib was more effective in the reverse scenario. This may simply reflect technical limitations of the smaller replicate numbers and shorter time-courses used in the chick embryo assay. However, it must also be noted that the mouse mammary fat pad study is more pathophysiologically relevant, as drug treatments were only started once tumors had reached a substantial (and standardized) volume. Since these model tumors were therefore more aggressive at the time of commencing treatment than the non-standardized ones in the chick embryo, it is reasonable to expect lower bioefficacy from a novel compound like JBS2, which works on a single new target protein, unlike lapatinib, which works on a very well-established drug target and has benefitted from huge investment over many years of pre-clinical optimization and clinical development.

Notwithstanding the value of both xenograft models discussed above, accumulating evidence favors a transition towards mouse models that inject tumor cells directly into mammary ducts (vs. the fat pad) to better recapitulate breast cancer microenvironments [15,36,37]. Direct intraductal approaches are particularly valuable for DCIS, offering not just a route for spatially accurate tumor seeding in the correct location where DCIS tumors form but also permitting precise local treatment of locally confined tumors [38]. SUM-225 cells have been demonstrated to form DCIS-like tumors in immunodeficient mice [36], and in our study, a variation of this model yielded intraductal tumors with high Ki67 positivity, whose bioluminescence was diminished by once-weekly treatment with peptide JBS2. The high significance of this finding is illustrated by the fact that JBS2 has a predicted half-life of only 30 h under mammalian in vitro conditions, but this is likely to be much shorter in vivo. It is also noteworthy that the short size of this peptide (five amino acids) makes it amenable to simple peptidomimetic conversion to improve its drug-like properties in future studies. Optimization could also reduce peptide doses required in future, as high micro-molar concentrations of JBS2 were necessary to produce biological effects in this study. Nonetheless, it was encouraging that even high concentrations of JBS2 were not systemically toxic in two mouse models—animals exhibited normal ambulatory and functional behaviors, and any mice lost during the course of treatment succumbed to known ailments of immunocompromised mice (e.g., lymphomas) rather than any obvious treatment-induced toxicity. Furthermore, no biochemical, hematological, or histopathological flags were raised in a prior pilot study of JBS2 tolerability in NOD-SCID mice. 

To begin interrogating possible signaling associated with JBS2-induced inhibition of tumor progression, two proteomic approaches were utilized. Tissues from both murine models were harvested at the end of treatment, and although temporal proteomic changes may have been missed, JBS2 induced more changes in the intraductal than the mfp model. Encouragingly, JBS2 reduced S241-phosphorylated levels of PDK1 relative to lapatinib treatment alone in mfp tumors. Since S241 phosphorylation of PDK1 is required for activation [39], and activated PDK1 phosphorylates the survival effector Akt [40], these data suggest that JBS2 might exert a better anti-survival effect than lapatinib in this model. This is supported by upregulation of the pro-apoptotic factor annexin A1 in mice treated intraductally with JBS2, but contradicted by evidence that levels of the anti-apoptotic protein HIAP2 were lowest in JBS2+lapatinib-treated animals in the mfp model. In the same model, it is also curious that levels of S241-phosphorylated PDK1 in lapatinib-treated animals exceeded those in PBS controls (or indeed JBS2 + lapatinib-treated animals), as lapatinib reportedly downregulates phospho-PDK1 (S241) in breast cancer cells [41]. This may be an early indicator of the development of therapeutic resistance, or perhaps JBS2-induced reductions in S241-phosphorylated PDK1 relate to a different downstream target of PDK1 (e.g., p70 ribosomal S6 kinase (S6K)). Levels of the S6K target S240-phosphorylated 40S-ribosomal protein-S6 (S6RIB) decreased modestly in JBS2 + lapatinib-treated extracts relative to PBS controls, but it is likely that alterations in phosphorylation of some targets were missed by the late-stage harvesting of tissues.

It was also encouraging that JBS2 reduced levels of activated (T180-phosphorylated) p38MAPK versus lapatinib (+/−JBS2) in the mfp model, since inhibition of MAPK activation reduces proliferation in ER-negative breast cancer cells [42] and can reverse EMT [43]. This fits with our finding that SPARC expression was upregulated by JBS2 in the intraductal model, potentially exerting a negative effect on tumor cell migration/invasion [44]. These physical characteristics are echoed to some extent in the cancer hallmark of angiogenesis, and JBS2 was found to increase the anti-angiogenic factor ADAM-TS1 in the intraductal model. However, caution is advised because although ADAM-TS1 was originally proposed as a tumor suppressor whose upregulation decreases growth in mammary tumor xenografts [45], high ADAM-TS1 expression has also been linked with the promotion of later metastasis in murine mammary cancer in vivo [46]. Similarly, this fits with tissues from JBS2-treated animals having increased levels of total but not activated (S338-phosphorylated) c-RAF, as c-RAF has been described to possess kinase-independent functions in promoting cell migration [47]. This does conflict with expectations that inhibiting JAM-A would reduce rather than increase cell migration [25,48], but balances against the fact that a lack of parallel enhancements in activated c-RAF would not drive a pro-proliferative phenotype in JBS2-treated animals. Furthermore, it illustrates the importance of not over-interpreting proteomic data at a late-stage endpoint that may not capture key pathways driving early DCIS development. While serial sampling would have been ideal to answer these questions, it was not possible under the ethical approval granted for this study. 

Less desirable proteomic results were that VEGFA, carbonic anhydrase IX (CAIX), and MUC16 protein levels all rose in mfp tumors treated with JBS2+lapatinib versus JBS2 alone. Changes in the former two proteins might imply a pro-angiogenic phenotype upon JBS2 and lapatinib co-treatment; indeed, there is evidence of VEGFA expression being associated with aggressive DCIS lesions [49], while high CAIX expression has been linked with a hypoxia-adaptive response in male DCIS [50]. Overexpression of MUC16, or the ovarian cancer antigen CA-125, in breast cancer has also been associated with neoplastic behavior [51]. However, it is notable that, in fact, ER-negative HER2-positive breast cancer patients with high gene expression of CAIX or MUC16 trended towards better recurrence-free survival intervals than those with low gene expression (Supplemental Figure S8). Therefore, the patient population represented by SUM-225 xenografts may benefit from drug-induced upregulation of these proteins. More concerning (at first glance) was the fact that TCL1A, FGFBP1, CD70, S100A4, TGFBR2, and CYR61 levels all increased in JBS2 versus PBS conditions for the intraductal model. Once again, closer examination revealed that high gene expression of 5/6 (excepting CYR61) trended towards better survival in ER-negative HER2-postitive breast cancer patients (Supplemental Figure S9). Consequently, it could be argued that JBS2 treatment was associated largely with positive proteomic indicators in both ER-negative HER2-positive mouse models used for this study. However, comprehensive proteomic studies will be important in future to begin unraveling the complex balance between pro- and anti-tumorigenic signals in JAM-A-antagonized tumor models.

It is reasonable to question the lack of overlap between targets altered by JBS2 treatment in the mfp versus intraductal models, but several differences may underlie the observed deviations. Firstly, mfp tumors were fresh-frozen, whereas intraductal tumors were FFPE, with false positives technically more likely in the latter. This would have been less of an issue had we used RPPA to directly compare the mfp versus intraductal tumors, but we used PEA technology, which was acknowledged by the manufacturer as being less suitable for FFPE material. Secondly, mfp tumors were effectively pure, since large (>100 mm^3^) volumes facilitated their direct excision and fixation. In contrast, intraductal tumors were smaller, with an unavoidable mix of mouse and human cells (estimated as 59 ± 1% human, *n* = 18 mice). Thirdly, intraductal mice could only be treated once-weekly to avoid ductal collapse. Therefore, intraductal JAM-A antagonism was significantly less than that in the mfp, in which mice were treated daily. Finally, although tissues from both models were harvested at 28 days for proteomic analysis, JBS2 treatment had lost its statistical significance against control tumors in the intraductal model by that time. This interval between 21 and 28 days, when significant functional differences were seen and then lost in the intraductal model, may have had a big impact on the upregulation or downregulation of proteins that are central to tumor progression pathways. It is therefore arguable that there may have been more overlap if proteomic analysis was conducted at 21 days in the intraductal model in parallel with 28 days in the mfp model. Notwithstanding these limitations, the identification of multiple statistically significant proteomic changes in both models will guide future studies on the signaling underlying JAM-A antagonism in breast cancer. Another limitation of this study was the fact that SUM-225 cells utilized in the three in vivo models represented only ER-negative HER2-positive tumors, thus providing no insight into the ~70% of DCIS tumors that are ER-positive [33]. However, since ER-negative HER2-positive DCIS lesions likely identify high-grade or recurrent disease [33], this study gives valuable insight into the proportion of DCIS tumors most urgently requiring drug targeting in patients. 

## 5. Conclusions

This study provides the first evidence that JAM-A is upregulated in human DCIS and can be successfully targeted in in vivo DCIS models to achieve functional reductions in tumor progression alongside signaling alterations which may themselves be pharmacologically targetable. The extracellular localization of the JAM-A *cis*-dimerization site makes it a readily targetable structure, and its spatial separation from the *trans*-dimerization site (which functions in physiological adhesion processes) offers assurance of the potential to selectively interrupt JAM-A function in cancer without impacting the important physiological functions of JAM-A. Furthermore the growing body of evidence for JAM-A overexpression on multiple tumor types [13] suggests that there may be broad applicability in its antagonism. Together with evidence that JAM-A loss in high-grade mammary tumor models promotes apoptosis [12], it is therefore timely and important to pursue deeper interrogation of JAM-A as a novel clinical target for preventing or treating early-stage HER2-positive ER-negative breast cancer.

## Data Availability

Data is contained within the article or Supplementary Materials and can be further discussed by contacting the corresponding author. The following publicly available dataset was interrogated during this study: https://kmplot.com/analysis/index.php?p=service&cancer=breast, accessed 8 October 2020.

## Supplementary Materials

The following supporting information can be downloaded at: https://www.mdpi.com/article/10.3390/cancers14051303/s1, Figure S1: Additive anti-viability effects of lapatinib and JBS2 in SUM-225 cells. (**A**) SUM-225 cells were plated at 4000 cells/well in technical triplicate in 6-well plates and treated with increasing concentrations of lapatinib (0–5 µM/L) or DMSO vehicle or 72 h in serum-containing medium before cell viability was assessed via MTT assay. The IC50 value was calculated as 0.13 µM/L using GraphPad Prism software. Graph represents mean ± SEM of *n* = 3 independent biological replicate experiments. (**B**) SUM-225 cells (2000/well) were plated as duplicate technical replicates in 96-well plates and treated with JBS2 (400 µg/mL) alone or in combination with the IC50 of lapatinib (0.1 µM/L) or vehicle control (PBS + DMSO) for 2 consecutive 72 h periods before cell viability was assessed using MTT assay. Graph represents mean ± SEM of 3 independent biological replicate experiments. There were statistically significant differences between vehicle and JBS2 (***), vehicle and lapatinib (**), or vehicle and JBS2+lapatinib (***); and between JBS2/lapatinib vs. either JBS2 (**) or lapatinib alone (**), where *p* < 0.01, *** *p* < 0.001 by one-way ANOVA. Figure S2: High expression of JAM-A in an LCIS tissue core. Membranous JAM-A immunohistochemical expression was semi-quantitatively scored in four LCIS cores within a commercial breast cancer tissue microarray. Representative low (4×) and high (20×) power images of JAM-A expression are shown for all four cores. One core was scored as 0 (**A**), one core was scored as 3+ across the vast majority of the section (**B**), and two cores were scored as 2+ in at least part of the sections (**C**). Figure S3: JBS2 reduces MCF7 cell viability and AKT signalling. (**A**) MCF7 cells were plated in triplicate wells (1500/well) of 96-well plates and treated 24 h later with 200, 400, or 800 μg/mL of JBS2 or an equivalent volume of PBS vehicle control. Cell viability was measured 72 h later by MTT assays. JBS2 reduced cell viability at both higher concentrations. Experiments were performed three times and data represent mean ± SEM, compared using two-tailed, equal variance Student’s *t*-tests, * *p* < 0.05. (**B**) MCF7 cells were plated in 6-well plates (150,000 per well) and treated 24 h later with 400 μg/mL of JBS2 or an equivalent volume of PBS. After 72 h, protein was extracted and analyzed by Western immunoblotting, essentially as described in Cruz et al. 2021 (PMID: 33669586; doi:10.3390/cancers13040871). Representative Western blot image and densitometric analysis of phosphorylated AKT (normalized to total AKT expression) and phosphorylated ERK1/2 (normalized to total ERK1/2 expression) revealed lower levels of pAKT and pERK1/2 expression following JBS2 treatment compared to control conditions. Experiments were performed three times and data represent mean ± SEM, compared using two-tailed, equal variance Student’s *t*-tests, * *p* < 0.05. The antibodies used for Western blotting were all sourced from Cell Signaling Technologies (Danvers, MA, USA) as follows: AKT (pan) (40D4; cat.# 2920), Phospho-AKT (Ser473; cat.# 9271), p44/42 MAPK (Erk1/2; cat.# L34F12), Phospho-p44/42 MAPK (Erk1/2; Thr202/Tyr204; cat.# 4370). Please also note that the PBS and JBS2 conditions for Supplementary Figure S3B were not run on adjacent lanes, so the cropped lane images were visually separated to ensure transparency. Figure S4: Generation of DCIS-like tumors in a mouse mfp xenograft model. SUM-225-luc cells (10^7^) were implanted into the mammary fat pads of NOD-SCID mice (without supplemental estrogen pellets) and immunohistochemically stained for JAM-A and HER2 when they reached a volume of ≥100 mm^3^. DCIS-like tumors formed principally within the ducts and featured strong membranous expression of both JAM-A and HER2. Figure S5: Lapatinib upregulated several key targets in reverse-phase protein array analysis of mfp tumors. Fresh-frozen tissues harvested from the SUM-225 mouse mammary fat pad model of breast cancer (treatment groups: PBS, JBS2, lapatinib, JBS2+lapatinib) were subjected to reverse-phase protein array (RPPA) proteomic analysis using a signaling-centered 55-protein array panel. Five protein targets were significantly upregulated by lapatinib treatment (Caspase 9 (D315), p53, Mcl1, p27, MAPK, * *p* < 0.05 by ordinary one-way ANOVA multiple comparisons test, using the original false discovery rate of Benjamini and Hochberg), confirming its success as a positive control for reducing tumor progression. Figure S6: JAM-A and HER2 antagonism evoke multiple proteomic changes in mfp tumor xenografts. Fresh-frozen tissues harvested from the SUM-225 mouse mfp model of breast cancer (treatment groups: PBS, JBS2, lapatinib, JBS2+lapatinib) were subjected to reverse-phase protein array (RPPA) proteomic analysis using a signaling-centered 55-protein array panel. Five individual protein targets changed significantly between JBS2 and any other condition in the 55-panel array (PDK1 (S241), p38MAPK (T180), cRAF, S6RIB (S240), HIAP2; * *p* < 0.05, ** *p* < 0.01; one-way ANOVA followed by Tukey’s HSD). Figure S7: JAM-A and HER2 antagonism had little effect on proteomic markers in SUM-225 cells. SUM-225 cells (2000/well) were plated as duplicate technical replicates in 6-well plates and treated with JBS2 (400 µg/mL) alone or in combination with the IC50 of lapatinib (0.1 µM/L) or DMSO vehicle control for two consecutive 72 h periods (treatment groups: PBS, JBS2, lapatinib, JBS2 + lapatinib). Protein extracts from *n* = 3 independent biological replicate experiments were then subjected to RPPA analysis using a signaling-centered 55-protein array panel. Only two individual protein targets changed significantly in this array (Src Y527 and Bcl-XL; * *p* < 0.05, ** *p* < 0.01 by ordinary one-way ANOVA multiple comparisons test, using the original false discovery rate of Benjamini and Hochberg). Figure S8: Gene expression profiles in ER-negative HER2-positive breast cancer patients. Gene expression levels of (**A**) CAIX (CA9), (**B**) MUC16, and (**C)** VEGFA were correlated with recurrence-free survival in ER-negative HER2-positive patients with the intrinsic genomic subtype of HER2 disease using an online Kaplan–Meier curve plotter tool (kmplot.com, accessed 8 October 2020). High levels of CAIX or MUC16 in this population trended towards better survival, while the opposite was the case for VEGFA. Figure S9: Gene expression profiles in ER-negative HER2-positive breast cancer patients. Gene expression levels of (**A**) TCL1A, (**B**) FGFBP1, (**C**) CD70 (CD27L), (**D**) S100A4 (P9KA), (**E**) TGFBR2, and (**F**) CYR61 were correlated with recurrence-free survival in ER-negative HER2-positive patients with the intrinsic genomic subtype of HER2 disease using the online Kaplan–Meier plotter (kmplot.com, accessed 8 October 2020). High levels of TCL1A, FGFBP1, CD70 (CD27L), S100A4 (P9KA), and TGFBR2 in this patient population trended towards better survival, while survival was unaffected by gene expression levels of CYR61. Table S1: Antibodies used for reverse phase protein array proteomic study. Table S2: Antibodies used for O-Link multiplex array proteomic study.
