# Supplementary material for: Functional Antagonism of Junctional Adhesion Molecule-A (JAM-A), Overexpressed in Breast Ductal Carcinoma In Situ (DCIS), Reduces HER2-Positive Tumor Progression
Source: Cancers (Basel). 2022 Mar 3;14(5):1303. doi: 10.3390/cancers14051303 (PMC8909510; doi:10.3390/cancers14051303)
Supplement: Supplementary file 1 [file cancers-14-01303-s001.zip › Smith et al_supplemental figs.pptx]

## Slide 1
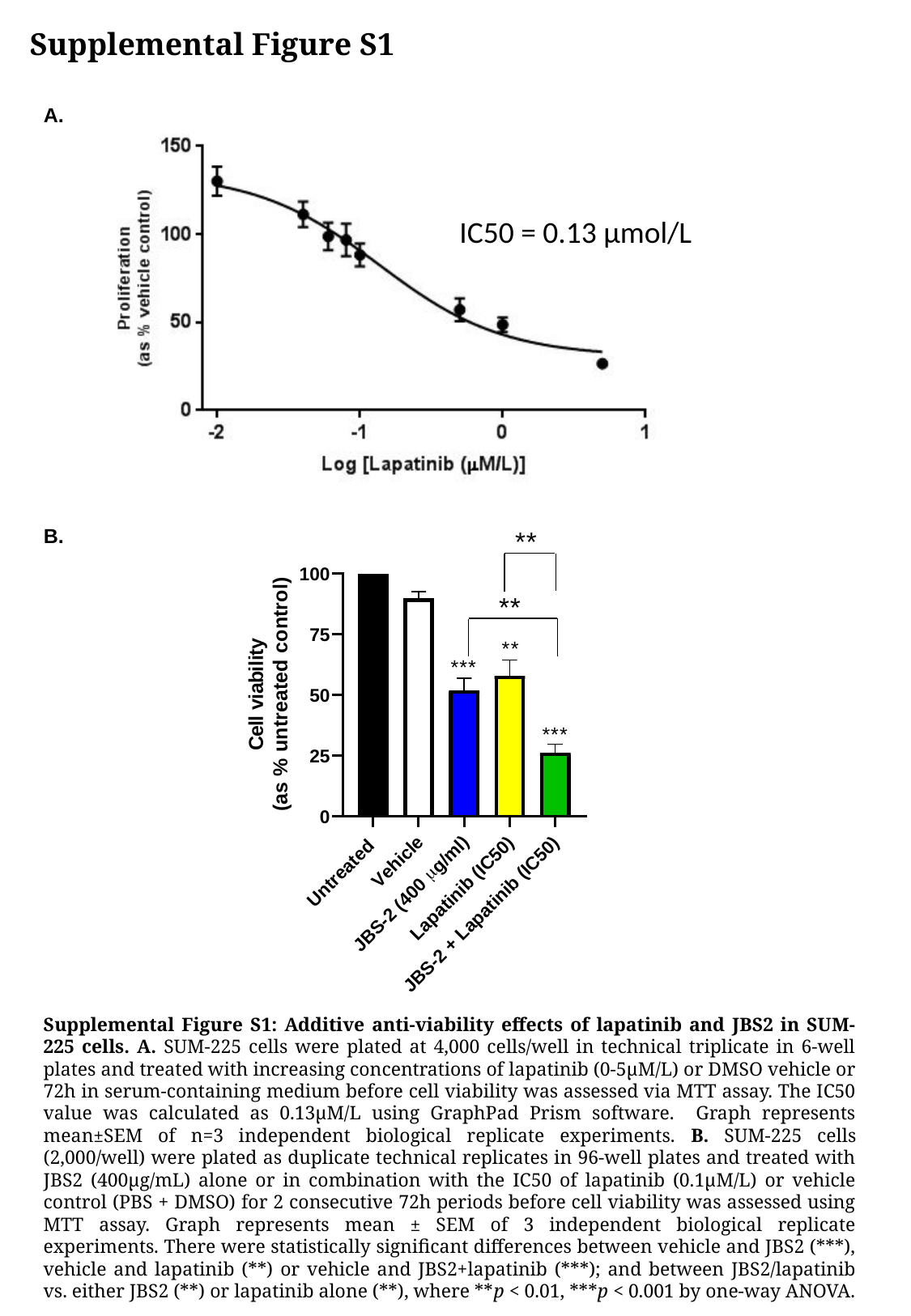

Supplemental Figure S1
A.
IC50 = 0.13 µmol/L
B.
Supplemental Figure S1: Additive anti-viability effects of lapatinib and JBS2 in SUM-225 cells. A. SUM-225 cells were plated at 4,000 cells/well in technical triplicate in 6-well plates and treated with increasing concentrations of lapatinib (0-5µM/L) or DMSO vehicle or 72h in serum-containing medium before cell viability was assessed via MTT assay. The IC50 value was calculated as 0.13µM/L using GraphPad Prism software. Graph represents mean±SEM of n=3 independent biological replicate experiments. B. SUM-225 cells (2,000/well) were plated as duplicate technical replicates in 96-well plates and treated with JBS2 (400µg/mL) alone or in combination with the IC50 of lapatinib (0.1µM/L) or vehicle control (PBS + DMSO) for 2 consecutive 72h periods before cell viability was assessed using MTT assay. Graph represents mean ± SEM of 3 independent biological replicate experiments. There were statistically significant differences between vehicle and JBS2 (***), vehicle and lapatinib (**) or vehicle and JBS2+lapatinib (***); and between JBS2/lapatinib vs. either JBS2 (**) or lapatinib alone (**), where **p < 0.01, ***p < 0.001 by one-way ANOVA.

## Slide 2
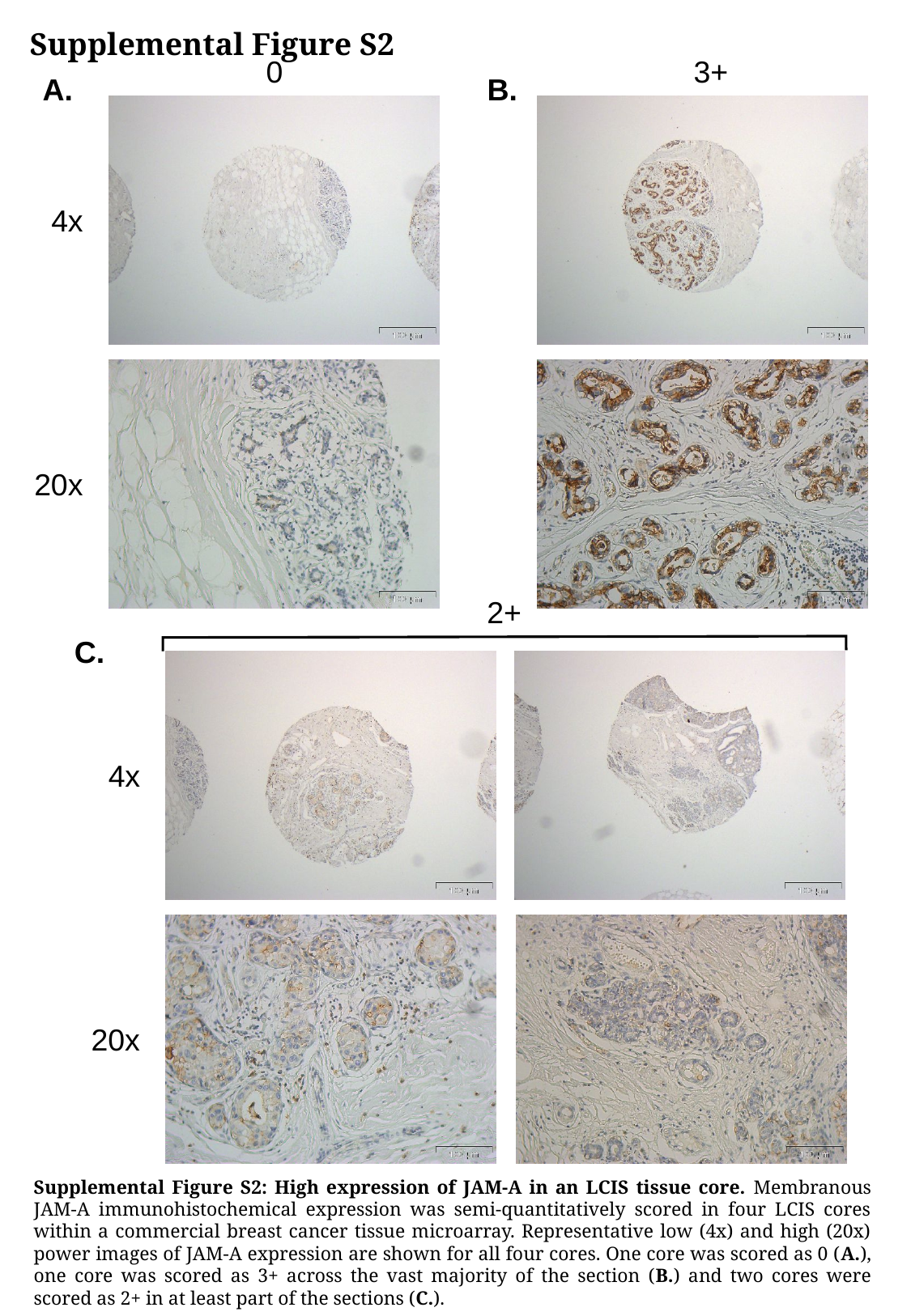

Supplemental Figure S2
0
3+
A.
B.
4x
20x
2+
C.
4x
20x
Supplemental Figure S2: High expression of JAM-A in an LCIS tissue core. Membranous JAM-A immunohistochemical expression was semi-quantitatively scored in four LCIS cores within a commercial breast cancer tissue microarray. Representative low (4x) and high (20x) power images of JAM-A expression are shown for all four cores. One core was scored as 0 (A.), one core was scored as 3+ across the vast majority of the section (B.) and two cores were scored as 2+ in at least part of the sections (C.).

## Slide 3
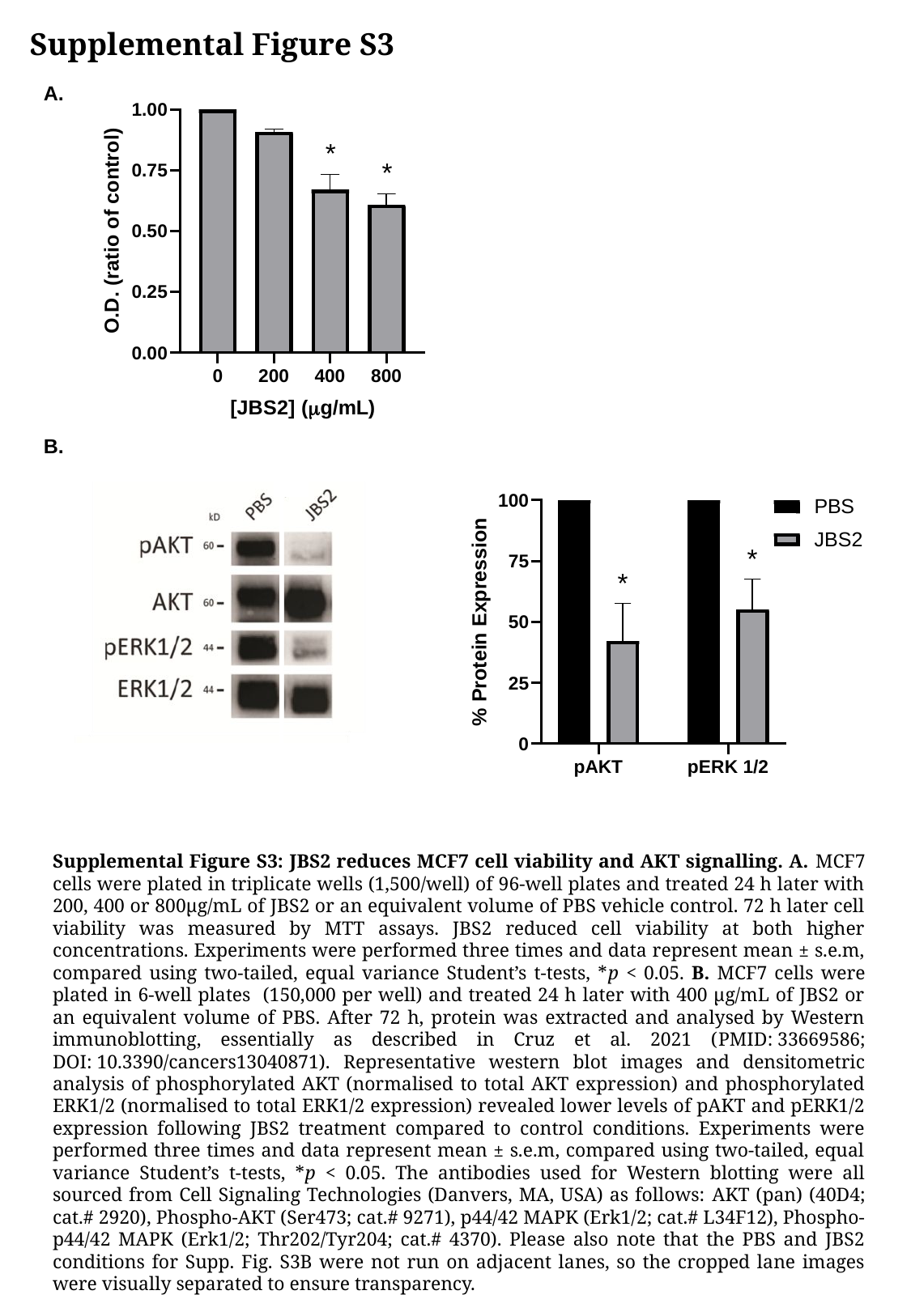

Supplemental Figure S3
A.
B.
Supplemental Figure S3: JBS2 reduces MCF7 cell viability and AKT signalling. A. MCF7 cells were plated in triplicate wells (1,500/well) of 96-well plates and treated 24 h later with 200, 400 or 800μg/mL of JBS2 or an equivalent volume of PBS vehicle control. 72 h later cell viability was measured by MTT assays. JBS2 reduced cell viability at both higher concentrations. Experiments were performed three times and data represent mean ± s.e.m, compared using two-tailed, equal variance Student’s t-tests, *p < 0.05. B. MCF7 cells were plated in 6-well plates (150,000 per well) and treated 24 h later with 400 μg/mL of JBS2 or an equivalent volume of PBS. After 72 h, protein was extracted and analysed by Western immunoblotting, essentially as described in Cruz et al. 2021 (PMID: 33669586; DOI: 10.3390/cancers13040871). Representative western blot images and densitometric analysis of phosphorylated AKT (normalised to total AKT expression) and phosphorylated ERK1/2 (normalised to total ERK1/2 expression) revealed lower levels of pAKT and pERK1/2 expression following JBS2 treatment compared to control conditions. Experiments were performed three times and data represent mean ± s.e.m, compared using two-tailed, equal variance Student’s t-tests, *p < 0.05. The antibodies used for Western blotting were all sourced from Cell Signaling Technologies (Danvers, MA, USA) as follows: AKT (pan) (40D4; cat.# 2920), Phospho-AKT (Ser473; cat.# 9271), p44/42 MAPK (Erk1/2; cat.# L34F12), Phospho-p44/42 MAPK (Erk1/2; Thr202/Tyr204; cat.# 4370). Please also note that the PBS and JBS2 conditions for Supp. Fig. S3B were not run on adjacent lanes, so the cropped lane images were visually separated to ensure transparency.

## Slide 4
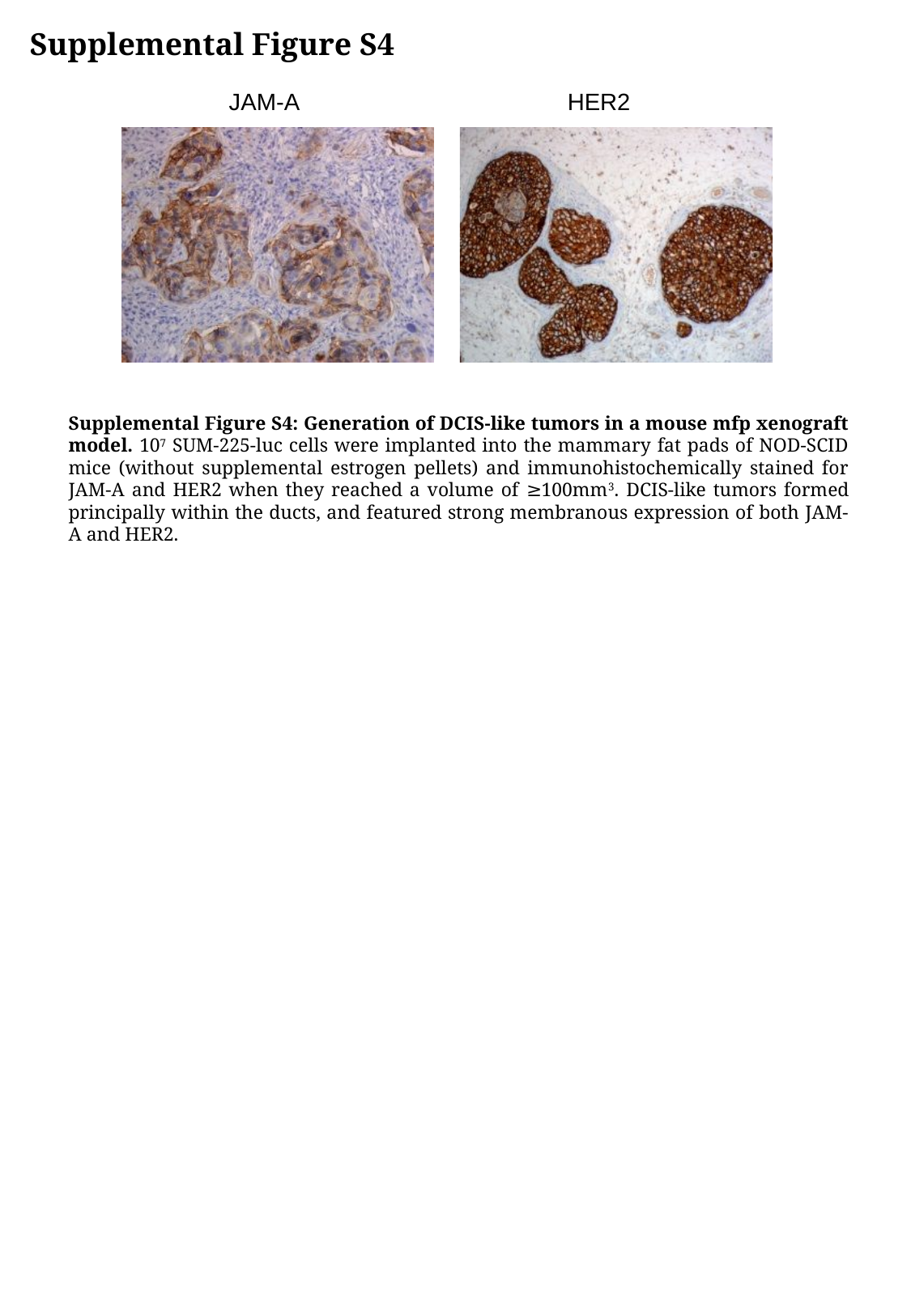

Supplemental Figure S4
JAM-A
HER2
Supplemental Figure S4: Generation of DCIS-like tumors in a mouse mfp xenograft model. 107 SUM-225-luc cells were implanted into the mammary fat pads of NOD-SCID mice (without supplemental estrogen pellets) and immunohistochemically stained for JAM-A and HER2 when they reached a volume of ≥100mm3. DCIS-like tumors formed principally within the ducts, and featured strong membranous expression of both JAM-A and HER2.

## Slide 5
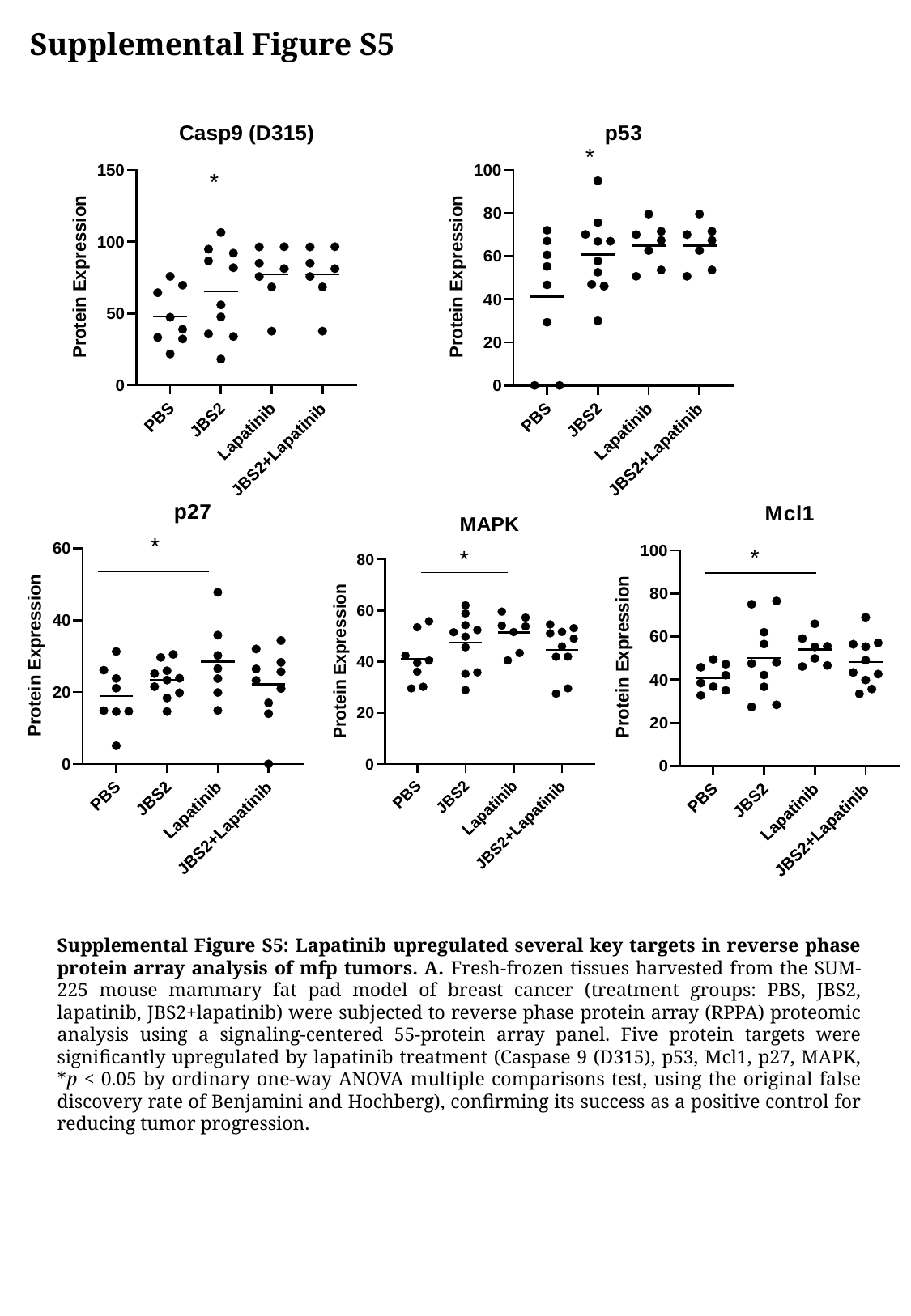

Supplemental Figure S5
*
*
*
*
*
Supplemental Figure S5: Lapatinib upregulated several key targets in reverse phase protein array analysis of mfp tumors. A. Fresh-frozen tissues harvested from the SUM-225 mouse mammary fat pad model of breast cancer (treatment groups: PBS, JBS2, lapatinib, JBS2+lapatinib) were subjected to reverse phase protein array (RPPA) proteomic analysis using a signaling-centered 55-protein array panel. Five protein targets were significantly upregulated by lapatinib treatment (Caspase 9 (D315), p53, Mcl1, p27, MAPK, *p < 0.05 by ordinary one-way ANOVA multiple comparisons test, using the original false discovery rate of Benjamini and Hochberg), confirming its success as a positive control for reducing tumor progression.

## Slide 6
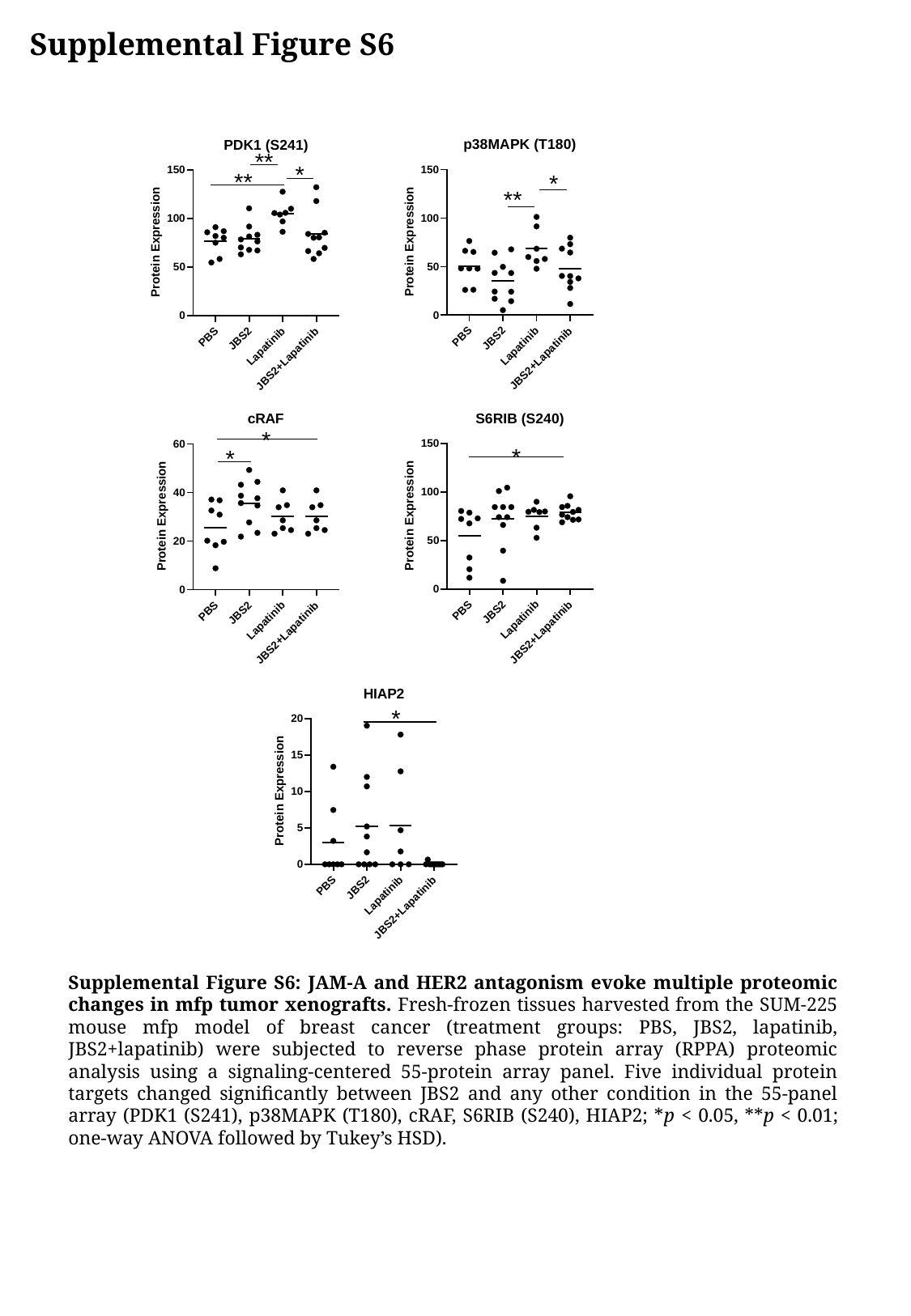

Supplemental Figure S6
*
**
**
*
**
*
*
*
*
Supplemental Figure S6: JAM-A and HER2 antagonism evoke multiple proteomic changes in mfp tumor xenografts. Fresh-frozen tissues harvested from the SUM-225 mouse mfp model of breast cancer (treatment groups: PBS, JBS2, lapatinib, JBS2+lapatinib) were subjected to reverse phase protein array (RPPA) proteomic analysis using a signaling-centered 55-protein array panel. Five individual protein targets changed significantly between JBS2 and any other condition in the 55-panel array (PDK1 (S241), p38MAPK (T180), cRAF, S6RIB (S240), HIAP2; *p < 0.05, **p < 0.01; one-way ANOVA followed by Tukey’s HSD).

## Slide 7
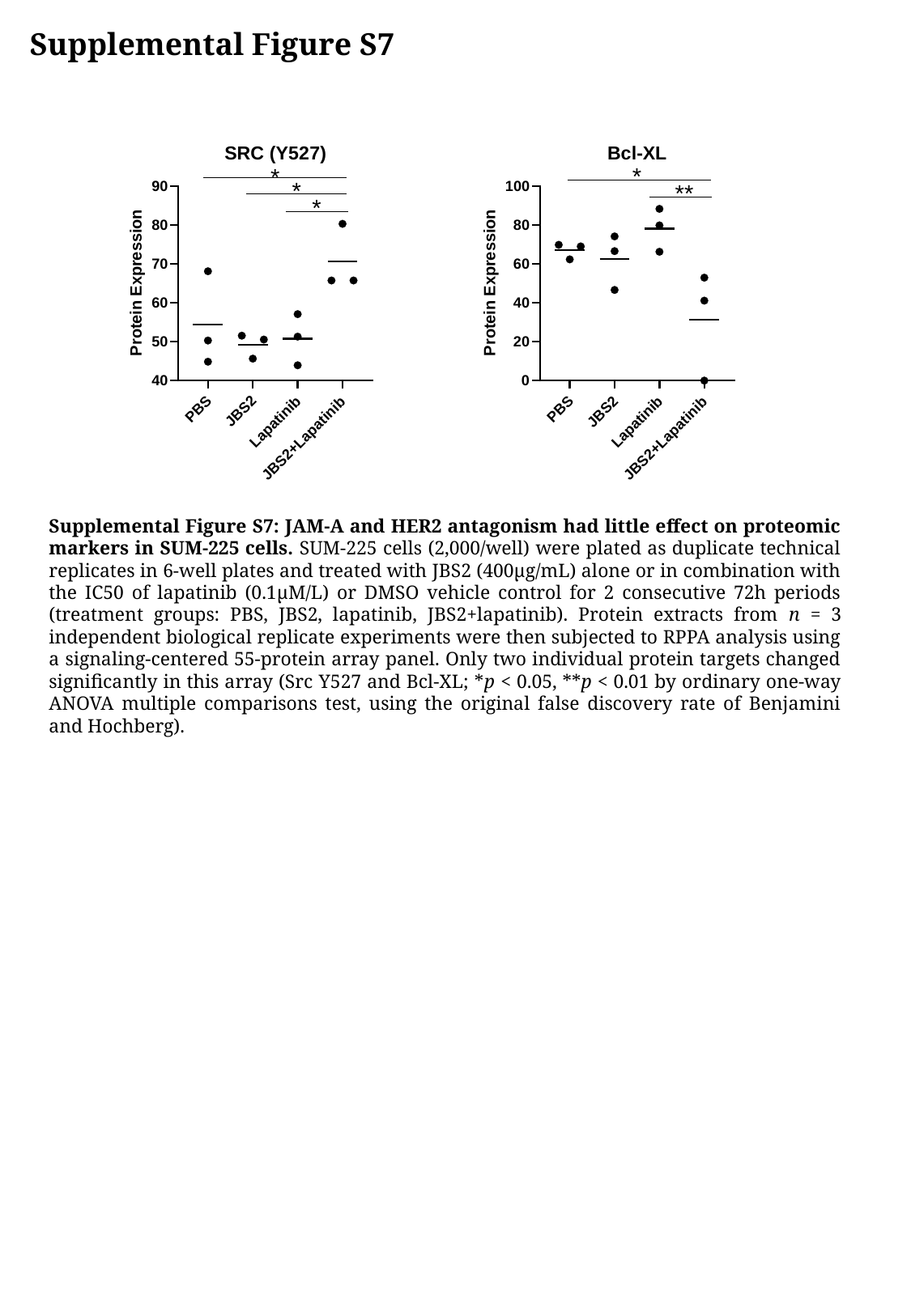

Supplemental Figure S7
*
*
*
**
*
Supplemental Figure S7: JAM-A and HER2 antagonism had little effect on proteomic markers in SUM-225 cells. SUM-225 cells (2,000/well) were plated as duplicate technical replicates in 6-well plates and treated with JBS2 (400µg/mL) alone or in combination with the IC50 of lapatinib (0.1µM/L) or DMSO vehicle control for 2 consecutive 72h periods (treatment groups: PBS, JBS2, lapatinib, JBS2+lapatinib). Protein extracts from n = 3 independent biological replicate experiments were then subjected to RPPA analysis using a signaling-centered 55-protein array panel. Only two individual protein targets changed significantly in this array (Src Y527 and Bcl-XL; *p < 0.05, **p < 0.01 by ordinary one-way ANOVA multiple comparisons test, using the original false discovery rate of Benjamini and Hochberg).

## Slide 8
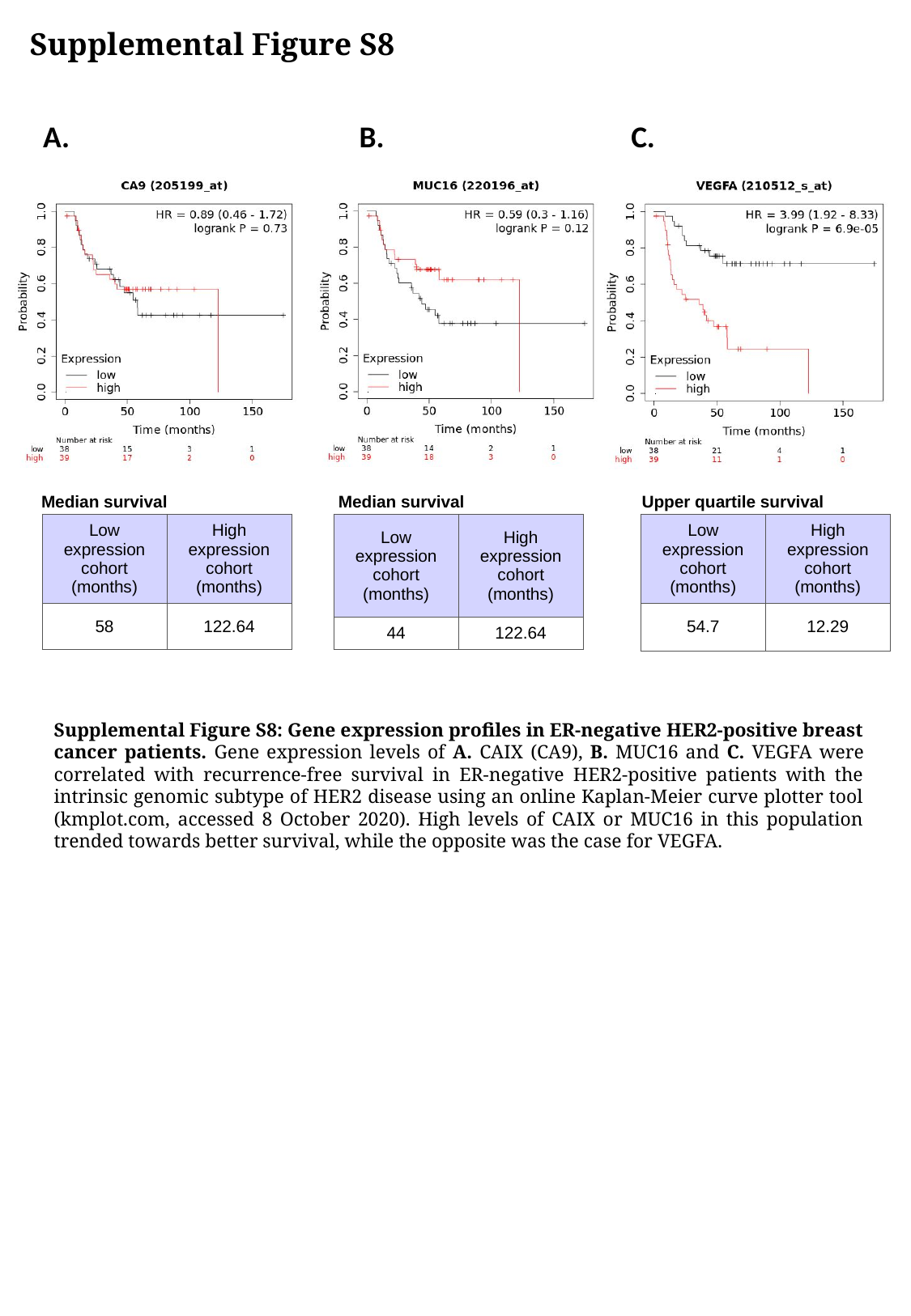

Supplemental Figure S8
A.
B.
C.
Median survival
Median survival
Upper quartile survival
| Low expression cohort (months) | High expression cohort (months) |
| --- | --- |
| 58 | 122.64 |
| Low expression cohort (months) | High expression cohort (months) |
| --- | --- |
| 44 | 122.64 |
| Low expression cohort (months) | High expression cohort (months) |
| --- | --- |
| 54.7 | 12.29 |
Supplemental Figure S8: Gene expression profiles in ER-negative HER2-positive breast cancer patients. Gene expression levels of A. CAIX (CA9), B. MUC16 and C. VEGFA were correlated with recurrence-free survival in ER-negative HER2-positive patients with the intrinsic genomic subtype of HER2 disease using an online Kaplan-Meier curve plotter tool (kmplot.com, accessed 8 October 2020). High levels of CAIX or MUC16 in this population trended towards better survival, while the opposite was the case for VEGFA.

## Slide 9
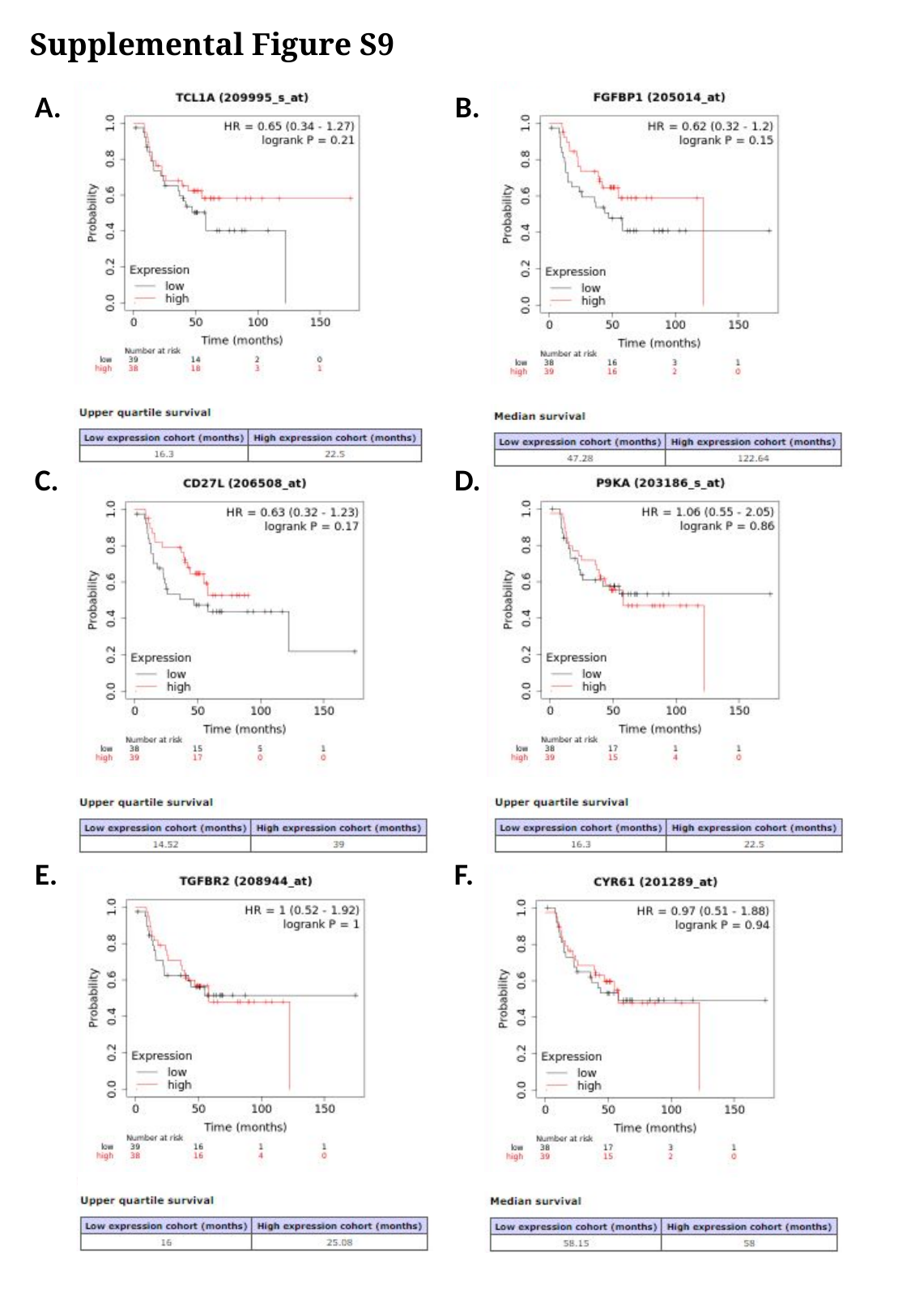

Supplemental Figure S9
A.
B.
C.
D.
E.
F.

## Slide 10
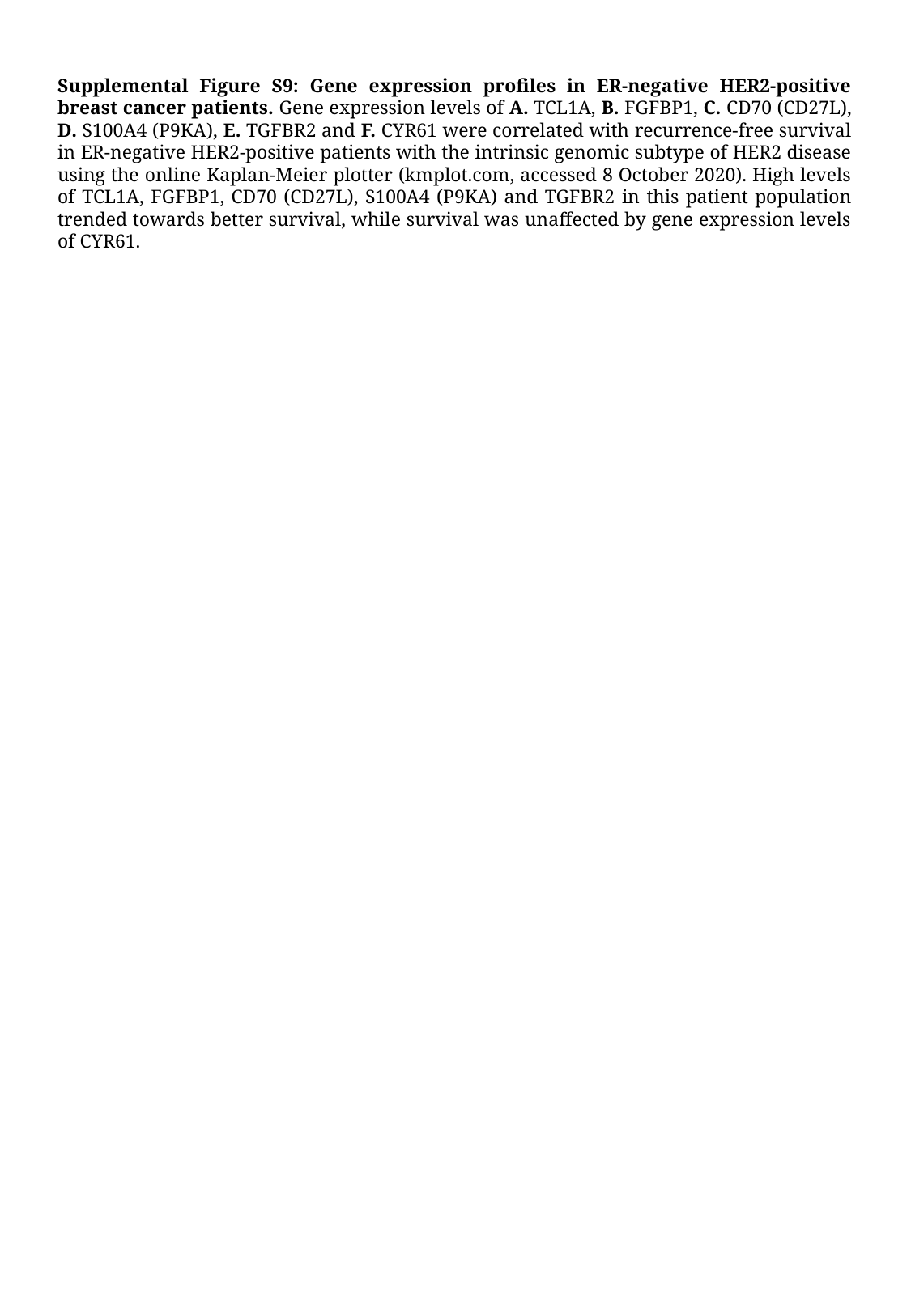

Supplemental Figure S9: Gene expression profiles in ER-negative HER2-positive breast cancer patients. Gene expression levels of A. TCL1A, B. FGFBP1, C. CD70 (CD27L), D. S100A4 (P9KA), E. TGFBR2 and F. CYR61 were correlated with recurrence-free survival in ER-negative HER2-positive patients with the intrinsic genomic subtype of HER2 disease using the online Kaplan-Meier plotter (kmplot.com, accessed 8 October 2020). High levels of TCL1A, FGFBP1, CD70 (CD27L), S100A4 (P9KA) and TGFBR2 in this patient population trended towards better survival, while survival was unaffected by gene expression levels of CYR61.
