# Supplementary material for: Functional Antagonism of Junctional Adhesion Molecule-A (JAM-A), Overexpressed in Breast Ductal Carcinoma In Situ (DCIS), Reduces HER2-Positive Tumor Progression
Source: Cancers (Basel). 2022 Mar 3;14(5):1303. doi: 10.3390/cancers14051303 (PMC8909510; doi:10.3390/cancers14051303)
Supplement: Supplementary file 1 [file cancers-14-01303-s001.zip › Smith_supplemental Tables.pdf]

Supplemental Table S1: Antibodies used for reverse phase protein array proteomic study

AKT	Mcl1
AKT(S473)	MEK1
AKT(T308)	MEK1_2(S217)
Akt2	MET
AMPK(T172)	MET(T1234)
AMPKalpha	mTOR
bak	mTOR(S2448)
Bax	NF_kB_p65
Bcl-xl	p27
Casp7_cleaved	p27(T157)
CASP9(D315)	p38MAPK
CASPASE8	p38MAPK(T180)
CHK1	p53
CHK1(S345)	PDK1
cRAF	PDK1(S241)
cRAF(S338)	PI3KalPha
EGFR	PKCalpha
FAK	PKCalpha(S657)
FAK(Y925)	PTEN
gab1(Y627)	S6RIB(S235)
GSK3B	S6RIB(S240)
GSK3B(S9)	SMAC
HER2	SRC
HIAP2	SRC(Y527)
IGFIR-beta	STAT3
LHDA	TIGAR
MAPK	VEFGR-2
MAPK(T202)	

Supplemental Table S2: Antibodies used for O-Link multiplex array proteomic study

5'-NT	FGF-BP1	PPY
ABL1	FR-alpha	PVRL4
ADAM 8	FR-gamma	RET
ADAM-TS 15	FURIN	RSPO3
ANXA1	Gal-1	S100A11
AREG	GPC1	S100A4
CAIX	GPNMB	SCAMP3
CD160	GZMB	SCF
CD207	GZMH	SEZ6L
CD27	HGF	SPARC
CD48	hK11	SYND1
CD70	hK14	TCL1A
CDKN1A	hK8	TFPI-2
CEACAM1	ICOSLG	TGF-alpha
CEACAM5	IFN-gamma-R1	TGFR-2
CPE	IGF1R	TLR3
CRNN	IL6	TNFRSF19
CTSV	ITGAV	TNFRSF4
CXCL13	ITGB5	TNFRSF6B
CXL17	KLK13	TNFSF13
CYR61	LY9	TRAIL
DLL1	LYN	TXLNA
EGF	LYPD3	VEGFA
EPHA2	MAD homolog 5	VEGFR-2
ERBB2	MetAP 2	VEGFR-3
ERBB3	MIA	VIM
ERBB4	MIC-A/B	WFDC2
ESM-1	MK	WIF-1
FADD	MSLN	WISP-1
FASLG	MUC-16	XPNPEP2
FCRLB	PODXL	
