# Supplementary material for: Functional Antagonism of Junctional Adhesion Molecule-A (JAM-A), Overexpressed in Breast Ductal Carcinoma In Situ (DCIS), Reduces HER2-Positive Tumor Progression
Source: Cancers (Basel). 2022 Mar 3;14(5):1303. doi: 10.3390/cancers14051303 (PMC8909510; doi:10.3390/cancers14051303)
Supplement: Supplementary file 1 [file cancers-14-01303-s001.zip › Smith_uncropped blots.pptx]

## Slide 1
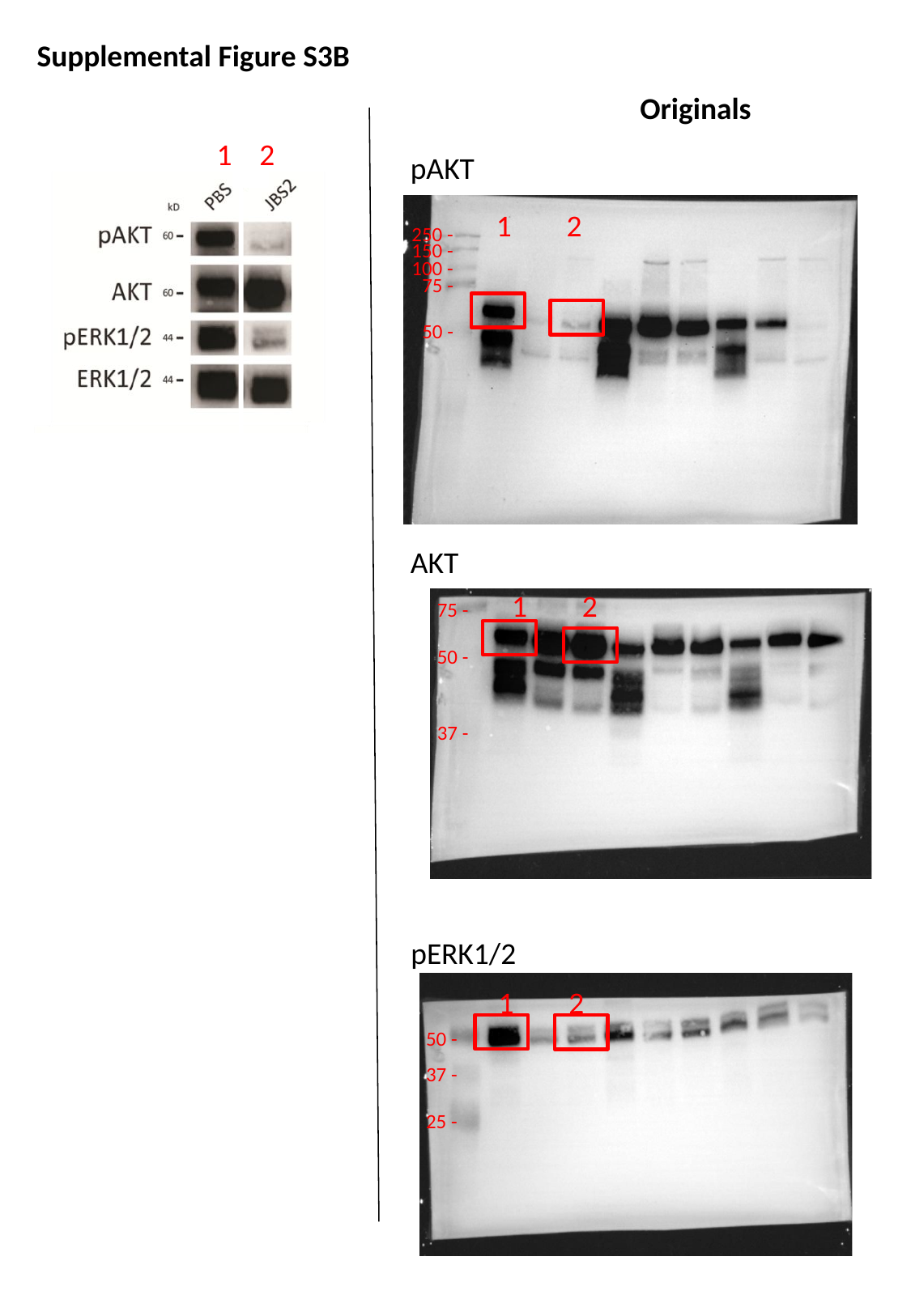

Supplemental Figure S3B
Originals
1 2
pAKT
1 2
250 -
150 -
100 -
75 -
50 -
AKT
1 2
75 -
50 -
37 -
pERK1/2
1 2
50 -
37 -
25 -

## Slide 2
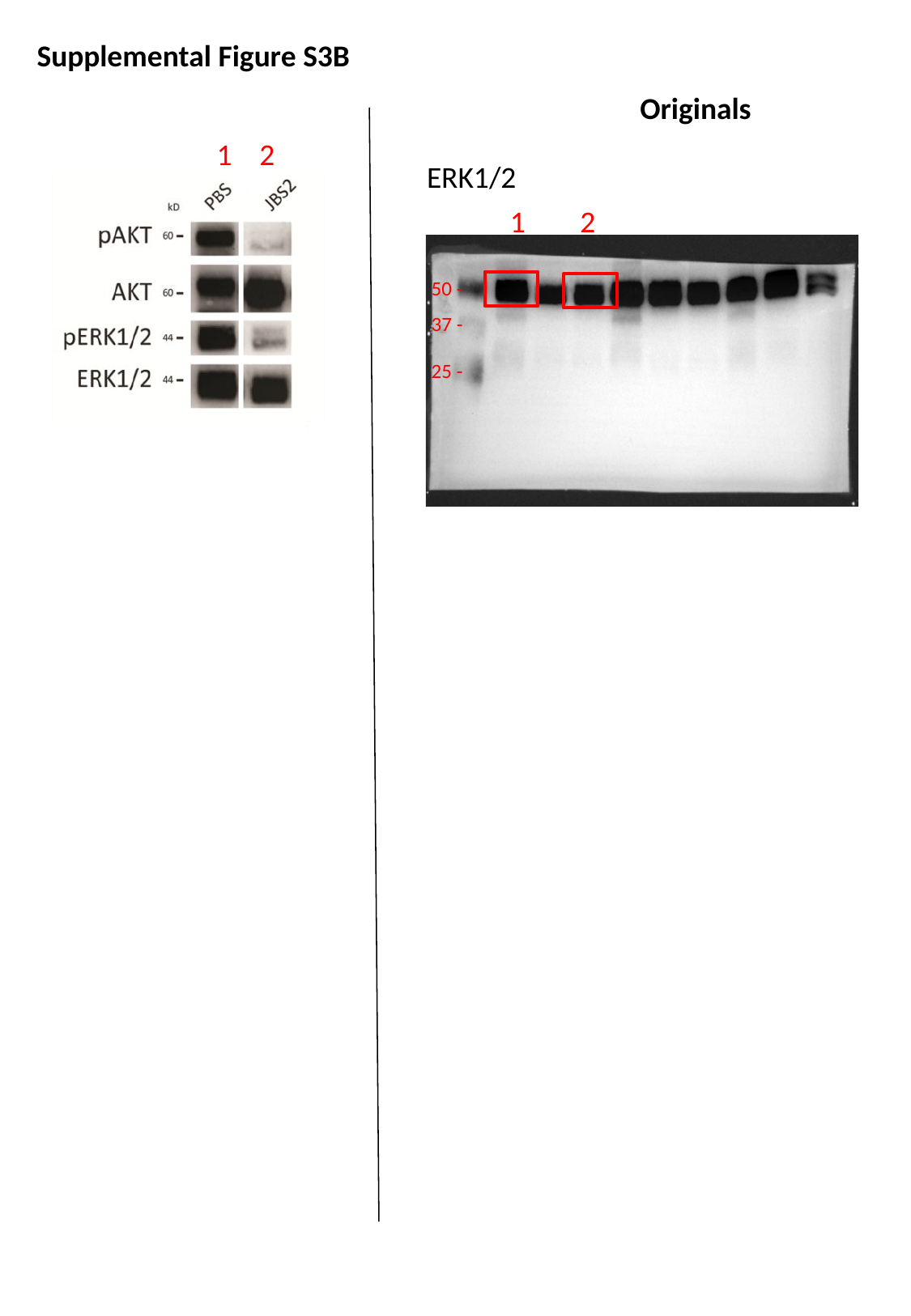

Supplemental Figure S3B
Originals
1 2
ERK1/2
1 2
50 -
37 -
25 -
